# Inhibition of SARS-CoV-2-Induced NLRP3 Inflammasome-Mediated Lung Cell Inflammation by Triphala-Loaded Nanoparticle Targeting Spike Glycoprotein S1

**DOI:** 10.3390/pharmaceutics16060751

**Published:** 2024-06-02

**Authors:** Chuda Chittasupho, Sonthaya Umsumarng, Kamonwan Srisawad, Punnida Arjsri, Rungsinee Phongpradist, Weerasak Samee, Wipawan Tingya, Chadarat Ampasavate, Pornngarm Dejkriengkraikul

**Affiliations:** 1 Department of Pharmaceutical Sciences, Faculty of Pharmacy, Chiang Mai University, Chiang Mai 50200, Thailand; chuda.c@cmu.ac.th (C.C.); rungsinee.p@cmu.ac.th (R.P.); wipawan_tingya@cmu.ac.th (W.T.); chadarat.a@cmu.ac.th (C.A.); 2 Faculty of Veterinary Medicine, Chiang Mai University, Chiang Mai 50100, Thailand; sonthaya.u@cmu.ac.th; 3 Center for Research and Development of Natural Products for Health, Chiang Mai University, Chiang Mai 50200, Thailand; 4 Department of Biochemistry, Faculty of Medicine, Chiang Mai University, Chiang Mai 50200, Thailand; kamonwan.sri@cmu.ac.th (K.S.); punnida_a@cmu.ac.th (P.A.); 5 Anticarcinogenesis and Apoptosis Research Cluster, Faculty of Medicine, Chiang Mai University, Chiang Mai 50200, Thailand; 6 Department of Pharmaceutical Chemistry, Srinakharinwirot University, Ongkharak, Nakhon Nayok 26120, Thailand; weerasak@g.swu.ac.th

**Keywords:** SARS-CoV-2, nanotriphala, nanoparticles, anti-inflammation, NLRP3 inflammasome pathway

## Abstract

The COVID-19 pandemic, caused by SARS-CoV-2, poses a significant global health threat. The spike glycoprotein S1 of the SARS-CoV-2 virus is known to induce the production of pro-inflammatory mediators, contributing to hyperinflammation in COVID-19 patients. Triphala, an ancient Ayurvedic remedy composed of dried fruits from three plant species—*Emblica officinalis* (Family Euphorbiaceae), *Terminalia bellerica* (Family Combretaceae), and *Terminalia chebula* (Family Combretaceae)—shows promise in addressing inflammation. However, the limited water solubility of its ethanolic extract impedes its bioavailability. In this study, we aimed to develop nanoparticles loaded with Triphala extract, termed “nanotriphala”, as a drug delivery system. Additionally, we investigated the in vitro anti-inflammatory properties of nanotriphala and its major compounds, namely gallic acid, chebulagic acid, and chebulinic acid, in lung epithelial cells (A549) induced by CoV2-SP. The nanotriphala formulation was prepared using the solvent displacement method. The encapsulation efficiency of Triphala in nanotriphala was determined to be 87.96 ± 2.60% based on total phenolic content. In terms of in vitro release, nanotriphala exhibited a biphasic release profile with zero-order kinetics over 0–8 h. A549 cells were treated with nanotriphala or its active compounds and then induced with 100 ng/mL of spike S1 subunit (CoV2-SP). The results demonstrate that chebulagic acid and chebulinic acid are the active compounds in nanotriphala, which significantly reduced cytokine release (IL-6, IL-1β, and IL-18) and suppressed the expression of inflammatory genes (*IL-6*, *IL-1β*, *IL-18*, and *NLRP3*) (*p* < 0.05). Mechanistically, nanotriphala and its active compounds notably attenuated the expression of inflammasome machinery proteins (NLRP3, ASC, and Caspase-1) (*p* < 0.05). In conclusion, the nanoparticle formulation of Triphala enhances its stability and exhibits anti-inflammatory properties against CoV2-SP-induction. This was achieved by suppressing inflammatory mediators and the NLRP3 inflammasome machinery. Thus, nanotriphala holds promise as a supportive preventive anti-inflammatory therapy for COVID-19-related chronic inflammation.

## 1. Introduction

The emergence of severe acute respiratory syndrome coronavirus 2 (SARS-CoV-2) and the ensuing coronavirus disease 2019 (COVID-19) pandemic have posed unprecedented challenges to global health systems. While the clinical spectrum of COVID-19 varies widely, ranging from asymptomatic cases to severe respiratory distress and long-term complications, a common thread underlying its pathogenesis is the dysregulated immune-inflammatory response triggered by viral infection [1,2]. Critical to the pathophysiology of COVID-19 is the pro-inflammatory milieu orchestrated by elevated levels of cytokines, including interleukin-6 (IL-6), interleukin-1β (IL-1β), and tumor necrosis factor-α (TNF-α), among others [3]. This cytokine storm contributes to systemic inflammation, vascular endothelial dysfunction, and tissue damage, culminating in acute respiratory distress syndrome (ARDS) and multiorgan dysfunction [4,5].

The intricate interplay between the Spike glycoprotein of SARS-CoV-2 and host cellular receptors, notably the angiotensin-converting enzyme 2 (ACE2) receptor, underscores the viral pathogenesis and immune response modulation [6]. Notably, the S1 subunit of the Spike protein, upon binding to ACE2, initiates a cascade of events leading to immune activation and inflammatory signaling [7,8]. Moreover, recent evidence implicates the activation of inflammasomes, particularly the nucleotide-binding oligomerization domain-like receptor containing pyrin domain 3 (NLRP3) inflammasome, in exacerbating COVID-19-associated inflammation [9,10,11].

In the pursuit of effective therapeutic strategies for COVID-19, there is burgeoning interest in harnessing the therapeutic potential of natural compounds with anti-inflammatory properties [12]. Triphala, an Ayurvedic formation comprising *Phyllanthus emblica* Linn., *Terminalia belerica* Linn., and *Terminalia chebula* Retz fruits, has garnered attention due to its diverse pharmacological properties, including anti-inflammatory effects [13,14,15,16]. Notably, polyphenolic constituents of Triphala such as gallic acid, chebulagic acid, and chebulinic acid, exhibit promising anti-inflammatory properties [17]. In arthritic rats, Triphala significantly reduced inflammatory cytokines and bone and cartilage degradation [18]. However, the translation of Triphala’s therapeutic benefits into clinical practice is hindered by challenges associated with its oral administration, particularly concerning bioavailability and patient compliance. Despite its therapeutic potential, the oral administration of Triphala at high doses poses challenges in terms of patient compliance. The dose of Triphala administered orally is very high, i.e., 2000 and 4000 mg of an aqueous extract formulation [19]. For the further application of Triphala extract for therapeutic purposes, the high concentration of Triphala ethanolic extract is difficult to dissolve in water (Table S1). To address this limitation, our study endeavors to formulate Triphala nanoparticles to enhance their solubility and therapeutic efficacy for use in applications such as oral administration and intranasal administration. By leveraging nanotechnology, we seek to optimize the delivery of Triphala’s bioactive compounds, thereby augmenting its anti-inflammatory effects and mitigating SARS-CoV2-induced inflammation.

Building upon previous investigations into nano formulations and natural compounds for COVID-19 therapy, our study aims to provide novel insights into the therapeutic potential of nanotriphala in managing inflammation associated with COVID-19. The nanoparticles carrying extracts of *Phyllanthus acidus* L. fruits, *Terminalia chebula*, and *Terminalia bellirica* also demonstrated antioxidant and anti-inflammatory properties in both in vitro and in vivo models [20,21,22,23]. While nano formulations and natural compounds have been explored individually in the context of viral infections and anti-inflammation, the synergistic combination of Triphala in nanoparticulate form targeting SARS-CoV-2-induced inflammation represents a novel approach [24,25,26]. In this study, we formulated nanoparticles containing Triphala ethanolic extract using the solvent displacement method to improve its solubility and therapeutic efficacy. Through comprehensive in vitro assessments of nanotriphala and its major constituents, we aim to elucidate their mechanisms of action in modulating inflammatory gene expression and cytokine release, with a specific focus on the NLRP3 inflammasome pathway.

Regarding the in vitro anti-inflammation experiment, many studies have adopted cell culture models using cell lines that express the angiotensin-converting enzyme 2 (ACE2) receptor such as type II pneumocyte cell line (A549 cells), human bronchial epithelial cells (HBEpCs), human nasal epithelial cells (HNECs), or the lung cancer cell line (Calu-3 cells) [8,27,28]. Therefore, in our investigation, the efficacy of the nanotriphala and its major constituents on attenuation of the SARS-CoV-2-induced inflammation on A549 lung cells was investigated. The findings from our study hold promise for the development of innovative therapeutic interventions to mitigate the inflammation sequelae of COVID-19.

## 2. Materials and Methods

### 2.1. Materials

The standard gallic acid, chebulagic acid, and chebulinic acid were obtained from MedChemExpress company (Monmouth Junction, NJ, USA). Poloxamer 407 was obtained from Chanjao Longevity Co., Ltd. (Bangkok, Thailand). Polyethylene glycol 400 was obtained from Dow Corporate Headquarters (Midland, MI, USA). A recombinant human coronavirus SARS-CoV-2 spike glycoprotein S1 (ab273068) was purchased from Abcam company (Cambridge, UK). Dulbecco’s Modified Eagle Medium (DMEM) was purchased from Gibco (Grand Island, NY, USA). The fetal bovine serum was purchased from Thermo Scientific Company (Waltham, MA, USA). The MTT or 3-(4,5-dimethylthiazol-2-yl)-2,5-diphenyltetrazolium bromide dye and mouse anti-beta-actin primary antibody were purchased from Sigma-Aldrich Company (St. Louis, MO, USA). Rabbit anti-NLRP3 primary monoclonal antibody, anti-ASC monoclonal antibody, anti-pro-caspase-1 (p50) monoclonal antibody, anti-cleaved-caspase-1 (p20) monoclonal antibody, and goat horseradish peroxidase-conjugated anti-mouse- or anti-rabbit-IgG were obtained from Cell Signaling Technology company (Danvers, MA, USA). The TRI reagent^®^ was purchased from Merck Millipore Company (Billerica, MA, USA). The ReverTra Ace^®^ qPCR Master Mix was purchased from Toyobo Co., Ltd. (Osaka, Japan). The SensiFAST SYBR Lo-ROX Kit was purchased from Meridian Bioscience^®^ Company (Cincinnati, OH, USA).

### 2.2. Triphala Extraction

The commercially available Triphala powder (mixture of dried and powdered fruits of three plants, *Emblica officinalis* (Family Euphorbiaceae), *Terminalia bellerica* (Family Combretaceae), and *Terminalia chebula* (Family Combretaceae) in equal proportions (1:1:1)) was obtained from Buanhuatuengherbs drug store in Phisanulok, Thailand (Thai traditional medicine license 27922). The powdered mixture was sieved through a sieve number 60. At room temperature, Triphala material (200 g) was macerated with 80% ethanol (2 L) under stirring for 24 h. The extract was filtered through filter paper to remove the marc. Triphala powder was repeatedly macerated one more time. After filtering the extracts, the extraction solvent was evaporated under reduced pressure with a rotary vacuum evaporator until the concentrated crude extract was obtained. The yield (%*w*/*w*) of the extract was calculated by the following equation.

$$Yield\left(\%\right)=\frac{Weight\;of\;extract}{Weight\;of\;dried\;powder}\times 100\%$$

### 2.3. Preparation and Characterization of Nanotriphala

Nanoparticles of Triphala extract were obtained using a solvent displacement method [29]. Briefly, Triphala ethanolic extract (500 mg) was dissolved in 1 mL of acetone and added dropwise (1 mL/h) into a mixture of 8 mL of deionized water and a stabilizer of 1% *w*/*v* poloxamer 407 (1 mL), and PEG 400 (1 mL) under magnetic stirring (600 rpm). The mixture was stirred overnight for 2 h to remove the organic solvent. Blank control was prepared without loading Triphala extract by infusing the organic solvent into a mixture of surfactant and stabilizer. The blank control was likely micelles formed when the PEO-PPO block copolymer concentration in solution reached a certain critical micellization concentration (CMC). At 30 °C, the CMC of poloxamer was 0.1%, which matches the concentration we used to prepare nanotriphala and blank control [30]. A dynamic light scattering (DLS) method was used to measure the size, polydispersity index value (PDI), and zeta potential of nanotriphala (5 mg/mL) at a 173° scattering angle and 25 °C (Zetasizer Nanoseries, Malvern Instruments, Malvern, UK).

### 2.4. Determination of the Characteristic and Physical Stability of Nanotriphala

The mean particle hydrodynamic diameter and polydispersity index of nanotriphala were assessed by dynamic light scattering (DLS) using a Zetasizer Nano Zs (Malvern, Worcestershire, UK) [31]. Zeta potential was determined using a Zetasizer Nano Zs with a laser Doppler anemometry (LDA)/phase analysis light scattering (PALS). The physical stability of nanotriphala was examined as a function of temperature and time. Nanotriphala (50 mg/mL) in deionized water was placed in a tight container and stored at 4, 30, and 45 °C for 3 months. A dynamic light scattering technique (Zetasizer ZS, Malvern, UK) was used to determine the hydrodynamic diameter, polydispersity value, and zeta potential value of the nanotriphala at certain time intervals (0, 1, 2, 3, and 4 months) to monitor the effect of the storage condition on the size, polydispersity, and surface charge of the nanotriphala.

### 2.5. Identification and Quantification of Bioactive Compounds in Nanotriphala Using High-Performance Liquid Chromatography (HPLC)

Identification of phytochemical content was confirmed by the HPLC technique, which was accomplished by comparing the retention times of relevant peaks to those of standard compounds. HPLC apparatus by Shimadzu (Prominence, Tokyo, Japan) equipped with a 250 mm × 4.6 mm (i.d.) reversed phase C18 column with 5 m particle size (Mightysil, Kanto Chemical Co., Tokyo, Japan) and a UV detector were used to determine the bioactive compound in nanotriphala. The separation condition of HPLC was modified according to a previous study [32]. The program of gradient elution is shown in Table 1. The flow rate was 1.0 mL/min, and the injection volume was 10 µL. A 0.45 µm membrane filter was used to filter samples before HPLC analysis. A UV detector was used to monitor the peaks at 295 nm. The amounts of gallic acid were calculated by the peak areas using a calibration curve constructed from 0.002 to 0.02 mg/mL of standard compounds. Unloaded nanoparticles were used as a reference. The standard gallic acid, chebulinic acid, chebulagic acid, ascorbic acid, maleic acid, ellagic acid, rutin, resveratrol, quercetin, kaempferol, 2,4-dihydroxy benzoic acid, catechin, epicatechin, and quercitrin were used in this study.

### 2.6. Determination of Total Phenolic Content in Nanotriphala

The total phenolic content in nanotriphala was quantified by the Folin–Ciaocalteau method. Gallic acid standard solution and nanotriphala (50 µL) were incubated with 10% *v*/*v* Folin–Ciaocalteu phenol reagent (100 µL). Then, 10% sodium carbonate solution (50 µL) was added to the mixture and incubated in the dark for 2 h at room temperature. The absorbance at a wavelength of 765 nm was read using a UV-visible spectrophotometer (Spectramax M3, Thermo Scientific, Waltham, MA, USA). The total phenolic content in nanotriphala was calculated from the gallic acid standard curve.

The encapsulation efficiency of nanotriphala was determined by measuring the total phenolic compounds in nanotriphala using the Folin–Ciocalteau method. Total phenolic contents were calculated from the gallic acid standard curve. Data were expressed as mg/g gallic acid equivalents of crude extract. The encapsulation efficiency was calculated by the following equation.

$$Encapsulation\;efficiency\left(\%\right)=\frac{Amount\;of\;total\;phenolic\;content\;in\;nanotriphala}{Initial\;amount\;of\;total\;phenolic\;content\;in\;triphala\;extract}\times 100\%$$

### 2.7. Release study of Nanotriphala

The release of total phenolic content from nanotriphala was performed by using Transwell^®^ chamber. Nanotriphala (50 mg/mL, 200 µL) was placed in the upper wells and the lower wells containing 1000 µL of PBS, pH 6.5. Then, Transwell was incubated at 37 °C. At time points of 0, 15 min, 30 min, 1, 2, 4, 6, 8, 12, and 24 h, samples (50 µL) were collected and diluted with DMSO (50 µL) for analysis and replaced with an equivalent volume of fresh PBS (50 µL). The cumulative release of total phenolic content from nanotriphala was measured at sampling time intervals by the Folin–Ciocalteau method and calculated by dividing the cumulative amount of TPC released by the amount of TPC loaded in the nanotriphala.

### 2.8. Chemical Stability Study of Nanotriphala

Nanotriphala were kept at 4, 30, and 45 °C and protected from light for 3 months. The sample was collected at 0, 1, 2, and 3 months to determine the total phenolic content by the Folin–Ciocalteau assay and was reported as % remaining of total phenolic compound in nanotriphala. The stability study of active compounds, gallic acid, chebulagic acid, and chebulinic acid in nanotriphala was determined using HPLC. Nanotriphala was stored under room temperature condition (30 °C). After 6 months, the concentration of gallic acid, chebulagic acid, and chebulinic acid in nanotriphala was analyzed by HPLC under the same conditions as the initial analysis.

### 2.9. Solubility Testing of Triphala Extract and Nanotriphala

Triphala extract and nanotriphala were weighed at 10 and 20 mg and were dissolved in different solvents: hexane, dichloromethane, ethyl acetate, ethanol, water, and dimethyl sulfoxide (DMSO) 1 mL. The solubility of Triphala extract and nanotriphala in each solvent was photographed and scored (+3 = fully soluble, +2 = partially soluble, +1 = slightly soluble, 0 = insoluble) at room temperature.

### 2.10. Cell Culture

The human lung epithelial cell line, A549 cells, was purchased from American Type Culture Collection (ATCC). The cell lines were cultured in DMEM supplemented with 10% FBS, 2 mM L-glutamine, 50 U/mL of penicillin, and 50 μg/mL of streptomycin. Cells were maintained in a 5% CO_2_ humidified incubator at 37 °C.

Primary human dermal fibroblasts were aseptically isolated from an abdominal scar after a surgical procedure involving a cesarean delivery at the surgical operation room of Chiang Mai Maharaj Hospital, Chiang Mai University, Chiang Mai, Thailand (Study code: BIO-2567-0035 approved by Medical Research Ethics Committee, Chiang Mai University). The fibroblasts were cultured in DMEM supplemented with 10% FBS, 2 mM L-glutamine, 50 U/mL penicillin, and 50 μg/mL streptomycin. Cells were maintained in a 5% CO_2_ humidified incubator at 37 °C.

### 2.11. Cytotoxicity Testing

The cytotoxicity of the nanotriphala, blank control, gallic acid, chebulagic acid, and chebulinic acid on A549 cells was determined using an MTT assay as has been previously described [33]. Briefly, the A549 cells (3 × 10^3^ cells/well) were seeded into a 96-well plate and incubated at 37 °C in 5% CO_2_ overnight. After that, the A549 cells were treated with increasing concentrations of nanotriphala, blank control (0–300 μg/mL), or gallic acid, chebulagic acid, and chebulinic acid (0–100 μg/mL) in a culture medium for 24 and 48 h. Then, the cells were incubated with 10 μL of 0.5 mg/mL MTT in PBS for 4 h. The culture supernatant was then removed, and the culture was re-suspended with 200 μL of DMSO to dissolve the MTT formazan crystals. The absorbance was measured at 540 and 630 nm using a UV-visible spectrophotometer. The assay was performed in triplicate at each concentration. Cell viability was calculated by comparing it with the control and interpreted as the % of control.

The cytotoxicity of the Triphala extract, nanotriphala, blank control, and gallic acid was confirmed using an SRB assay as previously described [34]. Briefly, A549 cells (3 × 10^3^ cells/well) were seeded in a 96-well plate and incubated at 37 °C, 5% CO_2_ overnight. After that, the cells were treated with or without various concentrations of nanotriphala, blank control (0–300 μg/mL), or gallic acid (0–50 μg/mL) in a culture medium for 24 and 48 h. Following incubation, the cells were treated with 10% (*w*/*v*) trichloroacetic acid (TCA) and incubated at 4 °C for 1 h. Subsequently, the medium was aspirated, and the cells were rinsed with gently running tap water. Next, 100 μL of a 0.054% (*w*/*v*) solution of sulforhodamine B (SRB) was added to each well, and the cells were incubated at room temperature for 30 min. After the incubation period, the SRB solution was removed, and the cells were washed four times with 1% (*v*/*v*) acetic acid. The cells were then allowed to dry at room temperature. To dissolve the dye, 150 μL of a 10 mM tris-based solution (pH 10.5) was added to each well, and the absorbance was measured at 510 nm using a microplate reader. The assay was performed in triplicate at each concentration. Cell viability was calculated by comparing it with the control and interpreted as the % of control.

### 2.12. Determination of Cytokine Secretion by ELISA Test

The cytokine secretions including IL-6, IL-1β, and IL-18 in cultured medium were examined using an ELISA kit (Biolegend, San Diego, CA, USA) and followed according to the manufacturer’s instruction. Firstly, A549 cells were seeded in a 6-well plate overnight. After that, the cells were pre-treated with nanotriphala (0–300 μg/mL), blank control (0–300 μg/mL), gallic acid (0–20 μg/mL), chebulagic acid (0–20 μg/mL), or chebulinic acid (0–20 μg/mL) for 24 h and then exposed to CoV2-SP at the concentration of 100 ng/mL for 3 h. The cultured medium was collected for ELISA testing, and the absorbance was measured at 450 and 570 nm. The cytokine releases were determined and calculated by comparing them with the standard curve.

### 2.13. Determination of IL-6, IL-1β, IL-18, and NLRP3 Genes by RT-qPCR Analysis

In order to determine inflammatory gene expressions (*IL-6*, *IL-1β*, *IL-18*, and *NLRP3*), A549 cells were pre-treated with nanotriphala (0–300 μg/mL), blank control (0–300 μg/mL), gallic acid (0–20 μg/mL), chebulagic acid (0–20 μg/mL), or chebulinic acid (0–20 μg/mL) for 24 h and then exposed to 100 ng/mL of CoV2-SP for 3 h. Then, the total mRNA was isolated using TRI reagent^®^. The concentration and purity of total RNA were detected using NanoDrop™ 2000/2000c Spectrophotometers (Thermo Fisher Scientific, Waltham, MA, USA). The cDNA was obtained via reverse transcription using a Mastercycler^®^ nexus gradient machine (Eppendorf SE, Hamburg, Germany). Then, a quantitative real-time PCR technique was determined using a qRT-PCR ABITM 7500 Fast & 7500 Real-Time PCR machine (Thermo Fisher Scientific, Waltham, MA, USA). Gene expressions were analyzed using QuantStudio 6 Flex real-time PCR system software v1.0 (Applied Biosystems, Waltham, MA, USA), and all primer sequences used in this study are shown in Table 2. Interleukin-6 (*IL-6*) primer sequence was supplied from Bio Basic Canada Inc., Markham, ON, Canada. Nucleotide-binding oligomerization domain-like receptor containing pyrindomain 3 (*NLRP3*), interleukin-1beta (*IL-1β*), interleukin-18 (*IL-18*), and glyceraldehyde 3-phosphate dehydrogenase (*GAPDH*) primer sequences were supplied from Humanizing Genomics Macrogen, Geumcheongu, Seoul, Korea. The 2^−ΔΔCT^ method with normalization to GAPDH and controls was used for the calculation of results.

### 2.14. Western Blot Analysis

To investigate the inhibitory mechanism of nanotriphala on inflammasome machinery proteins in CoV2-SP-induced inflammation, A549 cells were pre-treated with nanotriphala (0–300 μg/mL), chebulagic acid (0–20 μg/mL), or chebulinic acid (0–20 μg/mL) for 24 h and then exposed to 100 ng/mL of CoV2-SP for 3 h. Then, cells were collected and lysed using RIPA buffer. The protein concentration was determined using the Bradford method. The whole-cell lysate was subjected to 12% SDS-PAGE. Separated proteins were transferred into nitrocellulose membranes. Membranes were blocked with 5% non-fat dried milk protein in 0.5% TBS-Tween. After that, the membranes were washed twice with 0.5% TBS-Tween. Then, membranes were further incubated overnight with the primary antibody at 4 °C. Next, the membranes were washed 5 times with 0.5% TBS-Tween followed by incubating with horseradish peroxidase-conjugated anti-mouse or rabbit-IgG depending on the primary antibody at room temperature for 2 h and were then washed 5 times with 0.5% TBS-Tween. Bound antibodies were detected using the chemiluminescent detection system and then exposed to the iBright™ CL-1500 imaging system (Thermo Fisher Scientific, Waltham, MA, USA). Equal values of protein loading were confirmed as each membrane was stripped and re-probed with an anti-β-actin antibody. Band density levels were analyzed using IMAGE J 1.410 software.

### 2.15. Statistical Analysis

All data are presented as mean ± standard deviation (mean ± S.D.) values. Prism version 8.0 software was used for statistical analysis using an independent *t*-test and one-way ANOVA with Dunnett’s test. Statistical significance was determined at * *p* < 0.05, ** *p* < 0.01, *** *p* < 0.001, and **** *p* < 0.0001.

## 3. Results

### 3.1. Triphala Extract Yield and Solubility

A quantity of 200 g of Triphala powder underwent extraction utilizing 80% ethanol, resulting in the isolation of 110.08 g of Triphala extract. The yield of Triphala extract from 80% ethanol was 55.04 % *w*/*w*. The solubility of Triphala extract was tested using various solvents, such as hexane, dichloromethane, ethyl acetate, ethanol, water, and dimethyl sulfoxide (DMSO). The results showed that Triphala extract is fully soluble in DMSO, slightly soluble in water and ethanol, and insoluble in hexane, dichloromethane, and ethyl acetate (Table S1). From these results, high concentrations of Triphala extract (10 and 20 mg/mL) have poor solubility in water. Subsequently, the Triphala extract was employed in the synthesis of Triphala extract nanoparticles via a solvent displacement method for subsequent analysis.

### 3.2. Mean Particle Size, Size Distribution, Surface Charge, and Solubility of Nanotriphala

**Figure 1:**
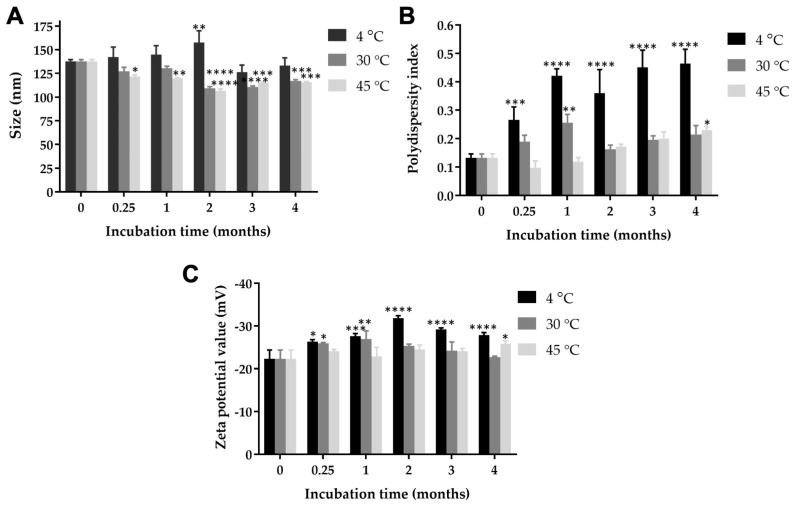
Physical characteristic of nanotriphala, (**A**) particle size, (**B**) polydispersity index, and (**C**) zeta potential of nanotriphala at various storage times (0.25, 1, 2, and 3 months) and temperatures (4 °C, 30 °C, and 45 °C). Particle size and polydispersity index were determined using dynamic light scattering, while zeta potential values were analyzed by measuring the electrophoretic mobility of nanotriphala. Data are presented as mean ± S.D. from three independent experiments. Statistical significance compared to freshly prepared nanotriphala is denoted as follows: * *p* < 0.05, ** *p* < 0.01, *** *p* < 0.001, and **** *p* < 0.0001.

Nanotriphala, nanoparticles derived from Triphala extract, were synthesized using the solvent displacement technique. The mean particle size, size distribution, and zeta potential values of nanotriphala were determined via dynamic light scattering techniques. Initially, the particle size of nanotriphala was measured to be 137.6 ± 1.9 nm. Following a storage period of 4 months, the hydrodynamic diameter of nanotriphala stored at 4 °C exhibited no significant change (133.1 ± 8.3 nm). Conversely, nanotriphala stored at 30 °C and 45 °C demonstrated a significant size reduction, measuring 116.9 ± 1.5 nm and 115.93 ± 0.4 nm, respectively (Figure 1A). The polydispersity index (PDI) of freshly prepared nanotriphala was 0.13 ± 0.01. However, after 4 months of storage, nanotriphala maintained at 4 °C, 30 °C, and 45 °C exhibited PDI values of 0.46 ± 0.05, 0.22 ± 0.03, and 0.23 ± 0.01, respectively (Figure 1B). These findings suggest a significant increase in PDI values for nanotriphala stored at 4 °C, likely attributed to the limited solubility of gallic acid and other phenolic compounds at lower temperatures. The zeta potential of nanotriphala remained unchanged after storage for 4 months at 30 °C (−22.73 ± 0.23 mV). Although the zeta potential of nanotriphala stored at 4 and 45 °C slightly increased, the values were in an acceptable range, i.e., −27.87 ± 0.59 mV and −25.77 ± 0.68 mV, respectively.

Furthermore, blank control displayed an average size of 179.37 ± 12.12 nm, with a PDI value of 0.33 ± 0.01, and a zeta potential value of −4.46 ± 0.94 mV. These results indicate that the encapsulation of Triphala extract into nanoparticles reduced both the hydrodynamic diameter and size distribution. Zeta potential measurements of nanotriphala ranged from −22.3 to −31.8 mV (Figure 1C). Notably, the increase in negative zeta potential values for nanotriphala compared to blank control suggests the presence of phenolic contents, the primary active compounds in Triphala extract. The negative surface charge of nanotriphala signifies nanoparticle stability.

The water solubility of nanotriphala compared to Triphala extract at the same concentrations is presented in Table S2. Nanotriphala at concentrations of 1, 5, 10, and 20 mg/mL is fully soluble in water, while Triphala extract is fully soluble in water only at a concentration of 1 mg/mL, partially soluble at 5 mg/mL, and slightly soluble at 10 and 20 mg/mL. Therefore, these findings indicate that nanotriphala significantly enhances the water solubility of Triphala extract.

### 3.3. Total Phenolic Content in Triphala Extract and Nanotriphala

**Figure 2:**
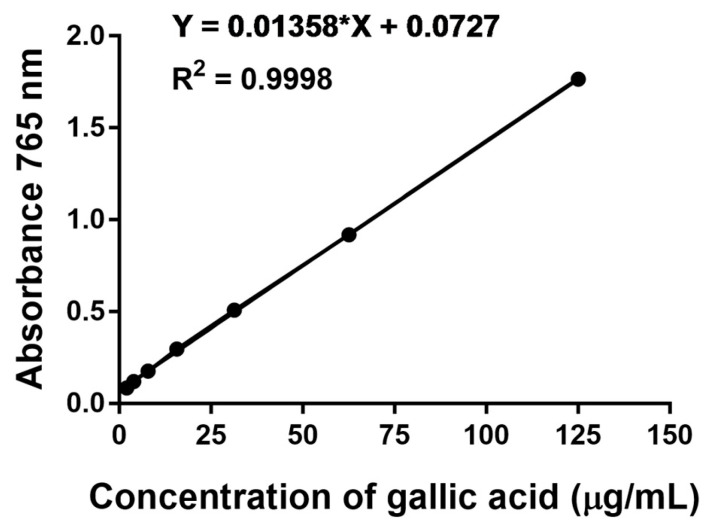
Folin–Ciocalteu assay standard curve. A standard curve depicting the relationship between concentration and absorbance in the Folin–Ciocalteu assay. The concentration of gallic acid equivalent was determined using the equation y = 0.0136x + 0.0727, with an r^2^ of 0.9998, where y represents absorbance and x represents the concentration of gallic acid. In the standard reaction, 50 µL of gallic acid concentration was mixed with 100 µL of Folin–Ciaocalteu phenol reagent and left to incubate at room temperature for 2 h. Then absorbance was subsequently measured at 765 nm. The resulting calibration curve was used to calculate the total phenolic content in nanotriphala.

Phenolic compounds are among the most effective constituents that contribute to anti-inflammatory activity. The total phenolic content of the ethanolic extract of Triphala was determined using a standard curve of gallic acid (y = 0.0136x + 0.0727; R^2^ = 0.9998) (Figure 2). The total phenolic content of Triphala ethanolic extract and nanotriphala was measured at 108.09 ± 2.80 mg GAE/g and 95.07 ± 2.81 mg GAE/g, respectively. The encapsulation efficiency of Triphala extract in nanotriphala was determined to be 87.96 ± 2.60%.

### 3.4. In Vitro Release Study

**Figure 3:**
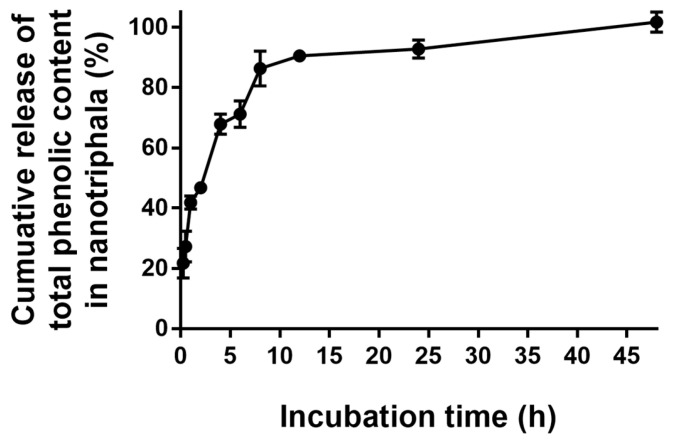
In vitro release profile of phenolic contents from nanotriphala. The graph depicts the in vitro release profile of phenolic contents from nanotriphala in phosphate buffered solution at pH 6.5 and 37 °C. Samples of nanotriphala were collected at various time intervals: 15 min, 30 min, 1 h, 2 h, 4 h, 6 h, 8 h, 12 h, 24 h, and 48 h. The total phenolic content was analyzed using the Folin–Ciocalteu method.

The in vitro release of total phenolic contents from nanotriphala was analyzed in PBS at pH 6.5, representative of the nasal cavity’s pH. Figure 3 illustrates the in vitro phenolic content release profile of nanotriphala. The data from the in vitro release study demonstrated that the cumulative amount of phenolic contents released from nanotriphala reached 92.79 ± 2.94% within 24 h and was fully released within 48 h (101.7 ± 3.3%). The high release rate was possibly due to the high solubility of phenolic content in the nanotriphala matrix. Analysis of the release kinetics revealed a biphasic release pattern, characterized by an initial burst release followed by a slower and more sustained release over 48 h. The initial burst release was observed within the first 8 h (86.3 ± 5.8%), indicating the presence of phenolic content primarily in the outer layer of nanoparticles. This phenomenon suggests the formation of a surfactant boundary layer on the surface of nanotriphala [36]. Kinetic modeling of the in vitro release data indicated that the release of phenolic content from nanotriphala at the initial burst release stage followed zero-order release kinetics, with an R^2^ value of 0.9306. These results suggest that the release rate of phenolic content from nanotriphala was independent of the remaining phenolic content within the nanoparticles.

### 3.5. Chemical Stability of Nanotriphala

**Figure 4:**
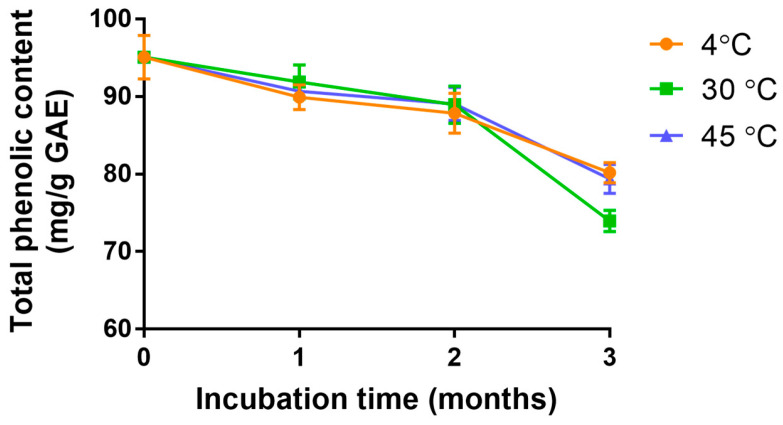
Long-term chemical stability prediction of nanotriphala. The figure illustrates the long-term chemical stability prediction of nanotriphala, extrapolated from accelerated stability data over a 3-month period of storage at temperatures of 4, 30, and 45 °C. The total phenolic content was quantified using the Folin–Ciocalteu method. Analysis of the determination coefficient (r^2^) indicated that the chemical degradation of nanotriphala followed zero-order kinetics.

The chemical stability of phenolic compounds in nanotriphala, when stored at 4 °C, 30 °C, and 45 °C, gradually decreased over a period of 3 months (Figure 4). The total phenolic content in nanotriphala reduced to 84.3%, 77.8%, and 83.5% when stored at 4 °C, 30 °C, and 45 °C, respectively. The linear regression analysis suggested that the degradation of phenolic content in nanotriphala followed zero-order kinetics, with R^2^ values of 0.9531, 0.8378, and 0.9035 at 4, 30, and 45 °C, respectively. Notably, the rate constant remained independent of storage temperature. The shelf life (the time for the drug to decay to 90% of its original concentration) of nanotriphala stored at 4, 30, and 45 °C was 2.14, 1.51, and 2.05 months, respectively. The stability of phenolic content was not significantly influenced by temperature variations. These findings suggest that phenolic compounds in Triphala within nanotriphala formulations exhibit stability even when stored at temperature as high as 45 °C.

### 3.6. Identification and Quantification of Bioactive Compounds in Nanotriphala Using High-Performance Liquid Chromatography (HPLC)

The Triphala extract was found to contain a high quantity of polyphenolic compounds, including gallic acid, chebulagic acid, and chebulinic acid [37,38]. To confirm the presence of these polyphenolic compounds in nanotriphala, the retention times of various compounds, including gallic acid, chebulinic acid, chebulagic acid, ascorbic acid, maleic acid, ellagic acid, rutin, resveratrol, quercetin, kaempferol, 2,4-dihydroxy benzoic acid, catechin, epicatechin, and quercitrin, were compared to those of standard compounds. In this study, the standard compounds were used as reference standards to construct the calibration curve for quantitation analysis. The HPLC chromatogram of reference standards and nanotriphala displayed in Figure S1. The results revealed gallic acid, chebulagic acid, and chebulinic acid as the predominant compounds in nanotriphala, present at concentrations of 1.19, 0.98, and 0.42 mg/100 mg, respectively. Additionally, significant amounts of gallic acid, chebulinic acid, chebulagic acid, ascorbic acid, and maleic acid were also identified, as detailed in Table 3. This HPLC analysis suggests the potential role of gallic acid, chebulagic acid, and chebulinic acid as active compounds in nanotriphala, contributing to their biological activity.

The stability study of active compounds in nanotriphala was performed by HPLC to quantify the concentration of gallic acid, chebulagic acid, and chebulinic acid in nanotriphala over a 6-month period. Samples were stored under room temperature condition. The results indicated a statistically significant reduction (*p* < 0.05) in the concentrations of gallic acid, chebulagic acid, and chebulinic acid over time (Figure S2). Specifically, gallic acid exhibited a 10% decrease, while chebulagic acid and chebulinic acid showed more pronounced reductions of approximately 40% and 65%, respectively. 

### 3.7. Effect of Nanotriphala and Its Bioactive Compounds on Cell Viability of A549 Cells

**Figure 5:**
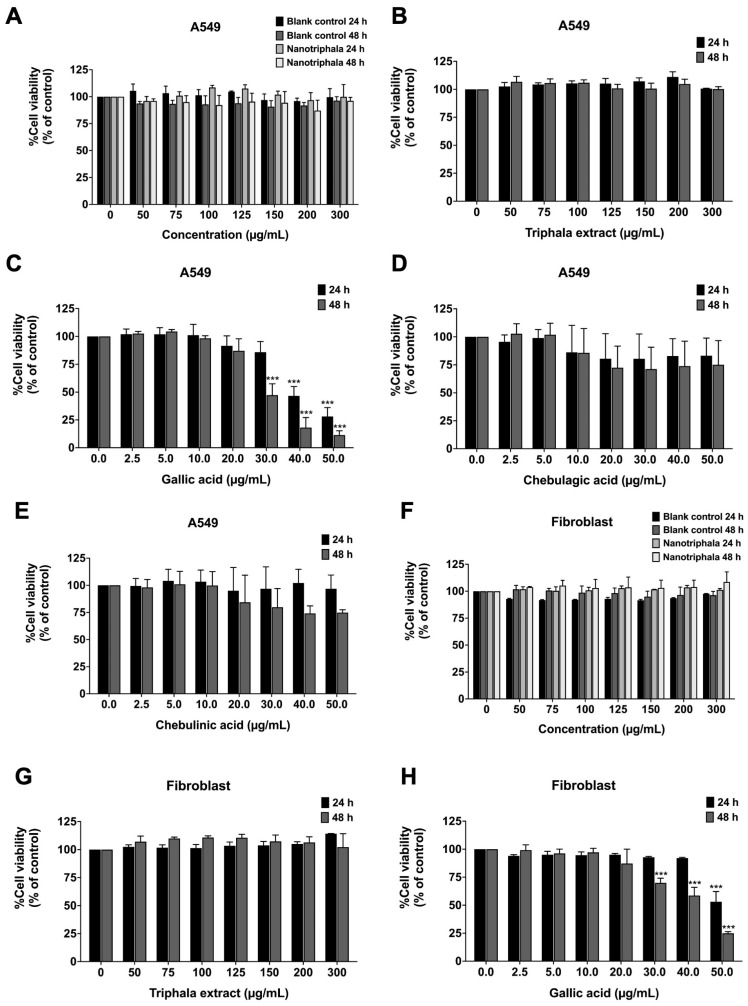
Cell viability of nanotriphala and blank control (**A**), Triphala extract (**B**), gallic acid (**C**), chebulagic acid (**D**), and chebulinic acid (**E**) on A549 cells. Cell viability of nanotriphala and blank control (**F**), Triphala extract (**G**), and gallic acid (**H**) on primary human dermal fibroblasts. Cells were treated with blank control, nanotriphala (0–300 μg/mL), and active compounds (gallic acid, chebulagic acid, and chebulinic acid) (0–50 μg/mL) for 24 and 48 h. Cell survival was determined using an MTT assay. Data are presented as mean ± S.D. values of three independent experiments, *** *p* < 0.001 compared with the control group.

Prior to assessing the anti-inflammatory properties of nanotriphala, we evaluated its impact on the cell viability of A549 cells using the MTT assay. Triphala extract, nanotriphala and blank control (Figure 5A,B), ranging in concentrations from 0 to 300 μg/mL, exhibited no significant cytotoxic effects on A549 cells (with an inhibitory concentration at 50% cell survival or IC_50_ > 300 μg/mL) when the A549 cells were pre-treated with nanotriphala and blank control for 24 h and 48 h, as shown in Figure 5A. Furthermore, chebulagic acid and chebulinic acid showed no cytotoxicity towards A549 cells following 24 h and 48 h incubation periods, as illustrated in Figure 5D and Figure 5E, respectively. In contrast, gallic acid exhibited a cytotoxic effect on A549 cells, with IC_50_ values of 28 μg/mL and 38 μg/mL after 24 and 48 h of incubation, as shown in Figure 5C. We have confirmed the cytotoxicity of Triphala extract, blank control, nanotriphala, gallic acid, chebulagic acid, and chebulinic acid on A549 cells using the SRB assay. Similar to the MTT assay, Triphala extract, blank control, nanotriphala (Figure S3A,B), chebulagic acid (Figure S3D), and chebulinic acid (Figure S3E) showed no cytotoxicity on A549 cells after 24 and 48 h of incubation. Gallic acid exhibited a cytotoxic effect on A549 cells, with IC_50_ values of 48 μg/mL and 37 μg/mL after 24 and 48 h of incubation, respectively, as shown in Figure S3C. Therefore, to investigate the anti-inflammatory properties of Triphala extract, nanotriphala and its three active compounds (gallic acid, chebulagic acid, and chebulinic acid) in A549 cells, we selected non-toxic concentrations of nanotriphala (0–300 μg/mL) and the three active compounds (0–20 μg/mL) for further experiments.

For safety purposes, we performed cytotoxicity tests of Triphala extract, blank control, nanotriphala, and gallic acid on normal cells. The cytotoxicity on normal human fibroblast was assessed using the MTT assay. The results showed that nanotriphala (Figure 5F) and Triphala extract (Figure 5G) at concentrations of 0–300 μg/mL did not induce cytotoxicity in primary human dermal fibroblasts after 24 or 48 h of treatment. Gallic acid, the major compound in nanotriphala that shows high cytotoxicity on A549 cells, also exhibited slight cytotoxicity on human dermal fibroblasts, with IC_50_ values of >50 μg/mL and 42 μg/mL after 24 and 48 h of incubation, respectively, as shown in Figure 5H. Thus, Triphala extract, blank control, nanotriphala, and gallic acid demonstrated no harmful effects on normal cells in the concentration range (0–20 μg/mL) used in this study.

### 3.8. Effects of Nanotriphala and Its Bioactive Compounds on the Inhibition of Pro-Inflammatory Cytokine Secretions in CoV2-SP-Induced A549 Cells

**Figure 6:**
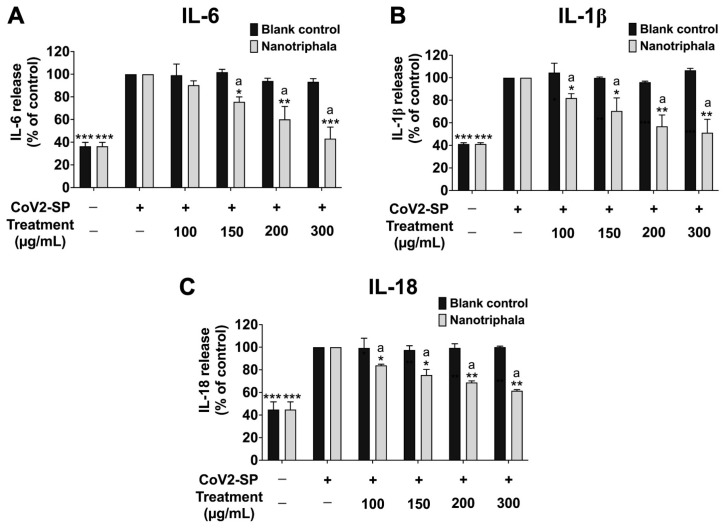
Inhibitory effects of nanotriphala and blank control on the pro-inflammatory cytokine secretion in CoV2-SP-induced A549 cells. A549 cells were pre-treated with nanotriphala and blank control (0–300 µg/mL) for 24 h. Then, the cells were exposed to CoV2-SP (100 ng/mL) for 3 h. The IL-6 (**A**), IL-1β (**B**), and IL-18 secretions (**C**) in the culture supernatant were examined by ELISA. The CoV2-SP-induced A549 cells are presented as 100%. Data are presented as mean ± S.D. values of three independent experiments, *** *p* < 0.001 compared with the CoV2-SP-induced control group. ^a^ * *p* < 0.05, ^a^ ** *p* < 0.01, ^a^ *** *p* < 0.001 compared with blank control at the same concentration.

To investigate the anti-inflammation effects of nanotriphala, the release of cytokines into the culture supernatant of CoV2-SP-exposed A549 cells was examined by ELISA testing. CoV2-SP exposure in A549 resulted in a significant increase in cytokine secretions of approximately 40% increase for IL-6, IL-1β, and IL-18 when compared with the non-CoV2-SP treated control group (Figure 6, *p* < 0.001). Pre-treatment of cells with nanotriphala at a concentration ranging from 100 to 300 µg/mL led to a dose-dependent decrease in the levels of IL-6, IL-1β, and IL-18 (Figure 6A–C), compared with the CoV2-SP-treated control group and each corresponding concentration of blank control (*p* < 0.05). To prove the efficacy of the nanotriphala formulation, the inhibitory effects of Triphala extract on cytokine release in CoV2-SP-exposed A549 cells were also examined and compared with nanotriphala using ELISA testing. The results showed that Triphala ethanolic extract and nanotriphala at the same concentration (300 µg/mL) did not show a statistically significant difference in the inhibition of pro-inflammatory cytokine secretions (IL-6, IL-1β, and IL-18), as shown in Figure S4.

**Figure 7:**
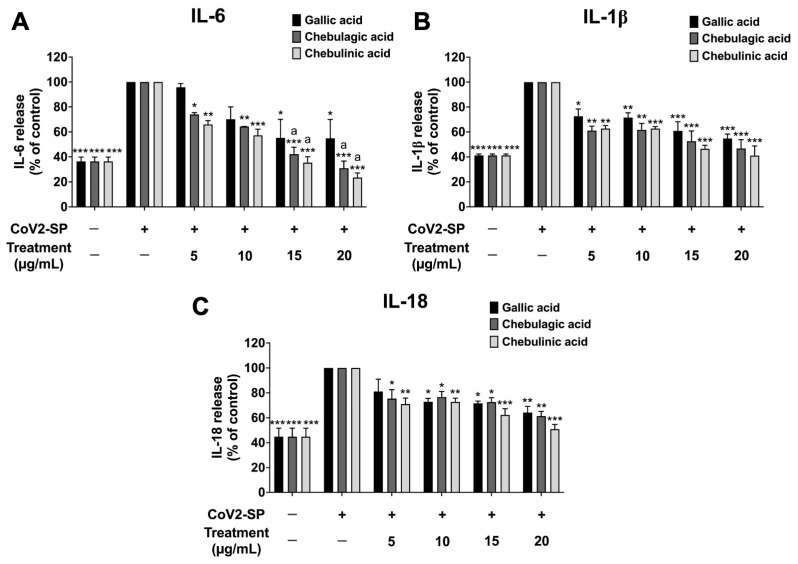
Inhibitory effects of active compounds (gallic acid, chebulagic acid, and chebulinic acid) on the pro-inflammatory cytokine secretion in CoV2-SP-induced A549 cells. A549 cells were pre-treated with active compounds (gallic acid, chebulagic acid, and chebulinic acid) (0–20 µg/mL) for 24 h. Then, the cells were exposed to CoV2-SP (100 ng/mL) for 3 h. The IL-6 (**A**), IL-1β (**B**), and IL-18 secretions (**C**) in the culture supernatant were examined by ELISA. The CoV2-SP-induced A549 cells are presented as 100%. Data are presented as mean ± S.D. values of three independent experiments, * *p* < 0.05, ** *p* < 0.01, and *** *p* < 0.001 compared with the CoV2-SP-induced control group. ^a^ *p* < 0.001 compared with gallic acid at the same concentration.

Regarding the three active compounds, treatment with gallic acid, chebulagic acid, and chebulinic acid significantly inhibited the release of IL-6, IL-1β, and IL-18 from CoV2-SP-induced A549 cells in a dose-dependent manner (*p* < 0.001) (Figure 7A–C). As shown in Figure 7A, a comparison of the inhibitory effects of chebulagic acid and chebulinic acid on pro-inflammatory cytokine release revealed that both compounds exhibited significantly more potent inhibition of IL-6 than gallic acid at concentrations of 15 or 20 μg/mL (*p* < 0.001).

### 3.9. Effects of Nanotriphala and Its Bioactive Compounds on the Inhibition of Pro-Inflammatory Gene Expressions in CoV2-SP-Induced A549 Cells

**Figure 8:**
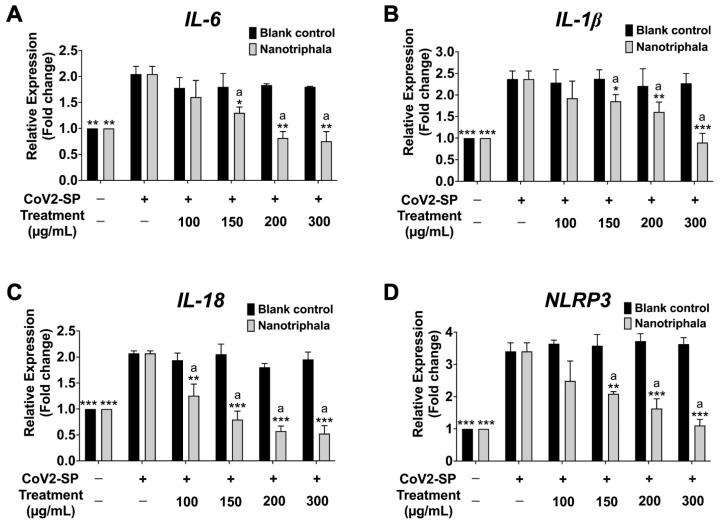
Inhibitory effects of nanotriphala and blank control on the *IL-6* (**A**), *IL-1β* (**B**), *IL-18* (**C**), and *NLRP3* gene expressions (**D**) in CoV2-SP-induced A549 cells. A549 cells were pre-treated with nanotriphala and blank control (0–300 µg/mL) for 24 h. Then, the cells were exposed to CoV2-SP (100 ng/mL) for 3 h. The mRNA expressions were determined using RT-qPCR. Data are presented as mean ± S.D. values of three independent experiments, ** *p* < 0.01 and *** *p* < 0.001 vs. the CoV2-SP-induced control group. ^a^ * *p* < 0.001 < 0.05, ^a^ ** *p* < 0.01, ^a^ *** *p* < 0.001 compared with blank control at the same concentration.

**Figure 9:**
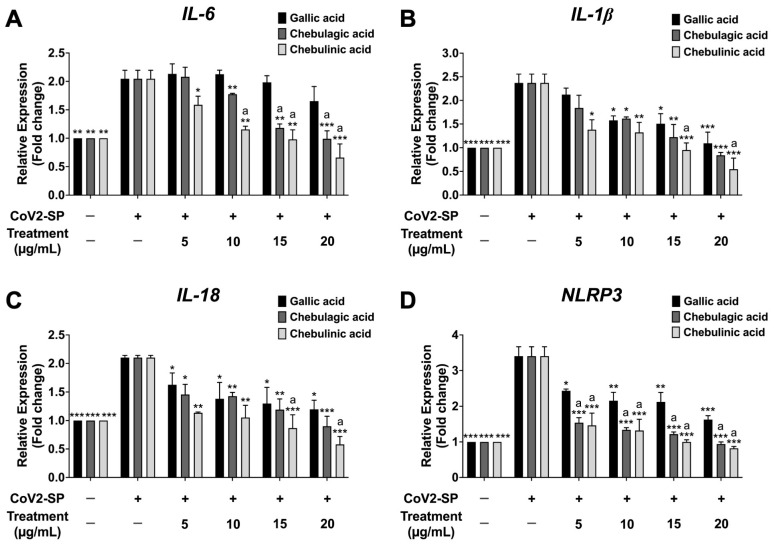
Inhibitory effects of active compounds (gallic acid, chebulagic acid and chebulinic acid) on the *IL-6* (**A**), *IL-1β* (**B**), *IL-18* (**C**), and *NLRP3* gene expressions (**D**) in CoV2-SP-induced A549 cells. A549 cells were pre-treated with active compounds (gallic acid, chebulagic acid and chebulinic acid) (0–20 µg/mL) for 24 h. Then, the cells were exposed to CoV2-SP (100 ng/mL) for 3 h. The mRNA expressions were determined using RT-qPCR. Data are presented as mean ± S.D. values of three independent experiments, * *p* < 0.05, ** *p* < 0.01 and *** *p* < 0.001 vs. the CoV2-SP-induced control group. ^a^ *p* < 0.001 compared with gallic acid at the same concentration.

To investigate the inhibition of inflammatory gene expression determined using reverse transcription-quantitative real-time polymerase chain reaction (RT-qPCR), CoV2-SP exposure in A549 resulted in a significant increase in mRNA levels as approximately 2-fold for *IL-6* and *IL-18*, 2.5-fold for *IL-1β*, and 3.5-fold for *NLRP3* when compared with the non-CoV2-SP treated control group (*p* < 0.01) (Figure 8). When the A549 cells were pre-treated with nanotriphala at the concentration of 100–300 µg/mL, the mRNA levels of those inflammatory genes were significantly decreased in a dose-dependent manner when compared with the CoV2-SP-treated control group and each blank control for the respective concentration of nanotriphala (*p* < 0.05). From the results, it can be concluded that nanotriphala possessed anti-inflammatory properties by inhibiting inflammatory gene expressions and inflammatory cytokine secretions. In addition, the inhibitory effects of Triphala extract on inflammatory genes expression in CoV2-SP-exposed A549 cells were also examined and compared with nanotriphala using the RT-qPCR technique. The results showed that Triphala ethanolic extract and nanotriphala at the same concentration (300 µg/mL) did not show a statistically significant difference in the inhibition of pro-inflammatory gene expressions (*IL-6*, *IL-1β*, *IL-18*, and *NLRP3*) in CoV2-SP-induced A549 cells, as shown in Figure S5. The three active compounds, gallic acid, chebulagic acid, and chebulinic acid treatment demonstrated pronounced inhibitory effects on *IL-6*, *IL-1β*, and *IL-18*, and *NLRP3* gene expressions in CoV2-SP-induced A549 cells in a dose-dependent manner (*p* < 0.05). As is shown in Figure 9A,D, chebulagic acid and chebulinic acid possessed significantly more potent decreased mRNA levels of *IL-6* and *NLRP3* inflammatory genes than gallic acid (*p* < 0.01).

Overall, it can be concluded that chebulagic acid and chebulinic acid (compared with gallic acid) exhibited greater anti-inflammatory properties upon CoV2-SP induction through a significant reduction in IL-6 cytokine releases and decreased inflammatory gene expression (*IL-6*, *IL-1β*, *IL-18*, and *NLRP3* genes). Therefore, we further investigated the inhibitory effects of nanotriphala and its active compound (chebulagic acid and chebulinic acid) on the NLRP3 inflammasome pathways against CoV2-SP-induced A549 cells.

### 3.10. Inhibitory Effects of Nanotriphala and Its Bioactive Compounds on NLRP3 Inflammasome Pathway in CoV2-SP-Induced A549 Cells

**Figure 10:**
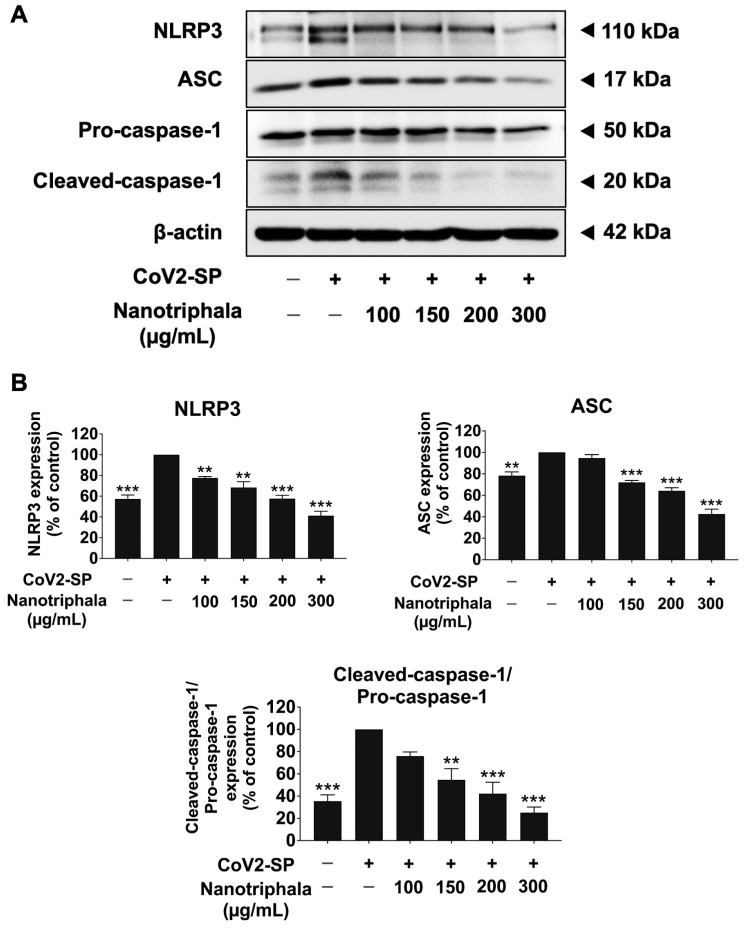
The effects of nanotriphala inhibited the NLRP3 inflammasome pathway in CoV2-SP-induced A549 cells. A549 cells were pre-treated with nanotriphala (0–300 µg/mL) for 24 h. Then, the cells were exposed to CoV2-SP (100 ng/mL) for 3 h. The inhibitory effects of nanotriphala on the expression of NLRP3, ASC, and pro-caspase-1 (p50) and cleaved-caspase-1 (p20) proteins in A549 cells are displayed in western blot (**A**) and band density measurements (**B**). The CoV2-SP-induced A549 is presented as 100% of control. Data are presented as mean ± S.D. values of three independent experiments, ** *p* < 0.01, and *** *p* < 0.001 compared with the CoV2-SP-induced A549 cells.

**Figure 11:**
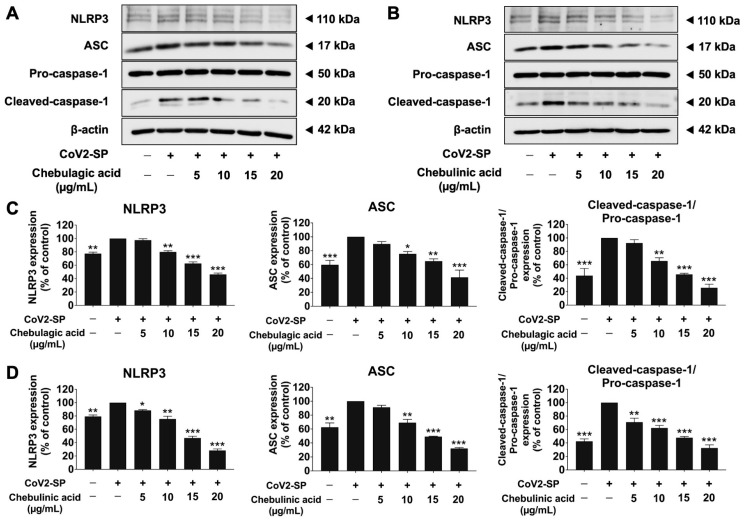
The effects of chebulagic acid and chebulinic acid inhibited the NLRP3 inflammasome pathway in CoV2-SP-induced A549 cells. A549 cells were pre-treated with chebulagic acid and chebulinic acid (0–20 µg/mL) for 24 h. Then, the cells were exposed to CoV2-SP (100 ng/mL) for 3 h. The inhibitory effects of chebulagic acid and chebulinic acid on the expression of NLRP3, ASC, and pro-caspase-1 (p50) and cleaved-caspase-1 (p20) proteins in A549 cells are displayed in western blot (**A**,**B**) and band density measurements (**C**,**D**). The CoV2-SP-induced A549 is presented as 100% of the control. Data are presented as mean ± S.D. values of three independent experiments, * *p* < 0.05, ** *p* < 0.01, and *** *p* < 0.001 compared with the CoV2-SP-induced A549 cells.

The NLRP3 inflammasome component comprises NLRP3, ASC, and pro-caspase-1 (p50). To activate the NLRP3 inflammasome, protein–protein interaction between NLRP3 and ASC causes the ASC to associate with pro-caspase-1 (p50). Then, pro-caspase-1 was activated to cleaved-caspase- 1 (p20), followed by the release of IL-1β and IL-18 cytokines. Therefore, we determined the inhibitory effects of nanotriphala and its active compound (chebulagic acid and chebulinic acid) on the NLRP3 inflammasome pathway using the western blot analysis. The result showed that CoV2-SP induction in A549 cells, the inflammasome machinery protein expressions, including NLRP3, ASC, pro-caspase-1 (p50), and cleaved-caspase-1 (p20) proteins, were significantly increased when compared with the non-CoV2-SP treated control (*p* < 0.01). When A549 cells pre-treated were with nanotriphala at the concentration of 100–300 µg/mL (Figure 10) and its active compounds at the concentration of 5–20 µg/mL (Figure 11), it was found that nanotriphala, chebulagic acid, and chebulinic acid could significantly decrease the CoV2-SP-induced inflammation via downregulating of inflammasome machinery proteins expressions (NLRP3, ASC, and Caspase-1) as well as the cleaved-caspase-1 expression in A549 cells in a dose-dependent manner (*p* < 0.05).

Overall, it can be concluded that nanotriphala and its active compound (chebulagic acid and chebulinic acid) were partially responsible for the anti-inflammatory properties upon CoV2-SP-exposed A549 cells via inhibition of the expressions of NLRP3, ASC, and pro-caspase-1 (p50) and the cleavage form of caspase-1 (p20), which would then lead to a decrease in pro-inflammatory cytokine releases (IL-1β and IL-18) at both the gene and protein levels.

## 4. Discussion

The outbreak of severe acute respiratory syndrome coronavirus 2 (SARS-CoV-2) since December 2019 has shown increasing severity and infectivity. The clinical symptoms that result in hospitalization primarily involve life-threatening acute respiratory syndrome and pulmonary deterioration, serving as the main clinical manifestations of severe COVID-19 [39,40]. These conditions typically occur in patients exhibiting elevated levels of pro-inflammatory cytokines, indicating that a cytokine storm may initiate the pathogenesis of the disease and the management of inflammation-related post-acute COVID-19 conditions, commonly known as Long-COVID [3,41]. The spike glycoprotein component of SARS-CoV-2 (CoV2-SP), an envelope glycoprotein aiding viral entry into host cells via immunogenic ACE2 receptor binding, represents a potential antiviral drug target [42]. Previous studies have provided evidence supporting the involvement of CoV2-SP in inducing inflammation during COVID-19. Furthermore, research has demonstrated that CoV2-SP can initiate an inflammatory response in various cell lines, including monocytes, macrophages, and lung epithelial cells [35,43,44,45]. In this study, we employed a cellular model to investigate CoV2-SP-induced inflammation through the NLRP3 inflammasome machinery and its cytokine products directly against A549 epithelial.

Triphala, an herbal formulation known for its various potential health benefits, contains a combination of three fruits (*Phyllanthus emblica* Linn., *Terminalia belerica* Linn., and *Terminalia chebula* Retz) with antioxidant, anti-inflammatory, anti-microbial, and anti-cancer properties [14,15,16,46]. However, the high concentration of Triphala ethanolic extract showed poor water solubility (Table S1), indicating a problem with bioavailability. Nanoparticle formulations are often used to enhance the solubility of poorly soluble compounds [47]. Therefore, in our study, nanotriphala was synthesized to improve the solubility of Triphala ethanolic extract while maintaining its anti-inflammatory properties. According to the literature, Triphala has never been formulated into a nanoparticulate delivery system despite its issues with water solubility. Huang et al. reported that Triphala aqueous extract was unstable due to its high polyphenol content, which caused sedimentation in the short term and reduced its efficacy [48]. It has been that the pH value of Triphala extract significantly influenced the association degree of polyphenols in the aqueous solution [49]. Increasing the pH value of Triphala extract can reduce the degree of polyphenol aggregation. However, increasing pH can lead to polyphenol chemical deterioration, such as catechin polymerization and changes in chemical composition. Huang et al. adjusted the pH value of the extract to 5.0 and demonstrated that sedimentation rates on the 3rd, 5th, 10th, and 15th days were reduced by 41%, 55%, 41%, and 23%, respectively [48]. In our study, the solubility of Triphala ethanolic extract was increased (Table S2) by using a solvent displacement method to form nanoparticles. This method reduced the particle size, resulting in a high surface area of nanostructured colloids, thereby increasing the extract’s solubility [50]. The nanoparticles were coated with a surfactant to enhance colloidal stability through steric stabilization [30].

Our study aimed to develop nanoparticles loaded with Triphala extract as a drug delivery system, termed nanotriphala. Nanoparticles of Triphala extract were obtained using a solvent displacement method, and we measured the size, polydispersity index value (PDI), and zeta potential of nanotriphala (5 mg/mL) at a 173° scattering angle and 25 °C using dynamic light scattering (DLS) method [31]. Our results suggested that nanotriphala had significantly larger polydispersity index values when stored at 4 °C, while the values of nanotriphala stored at 30 and 45 °C were not changed. PDI is another important parameter describing the width or spread of the particle size distribution. The increase in the polydispersity index value of nanotriphala stored at 4 °C might be due to the limited water solubility of gallic acid and other phenolic compounds at low temperatures. Further, the increase in the polydispersity index of nanotriphala might result from the dehydration and dense PPO block of poloxamer, while the overall size of nanoparticle grew slightly [30]. In addition, properties such as the viscosity of the medium may affect micelle shape and polydispersity index [51]. By providing detailed insights into the properties of nanotriphala, our study contributes to understanding its potential efficacy and mechanism of action.

Triphala is rich in polyphenolic compounds such as gallic acid, ellagic acid, chebulinic acid, bellericanin, β-sitosterol, and flavonoids. Our results revealed that the total phenolic content of Triphala ethanolic extract and nanotriphala was found to be 108.09 ± 2.80 mg GAE/g and 95.07 ± 2.81 mg GAE/g, respectively. The encapsulation efficiency of Triphala extract in nanotriphala was 87.96 ± 2.60%. Previous studies have reported varying phenolic contents in Triphala extracts obtained using different solvents. The ethanolic extract of Triphala was found to be 43.36 ± 0.10 mg/g GAE of phenolic content as reported by Malik et al., whereas Triphala extracted from water, methanol, and acetone contained 66.24, 71.97, and 38.44 mg/g GAE [52]. Vadde et al. reported that ethanolic and aqueous extracts of Triphala possessed about 180 mg/g GAE and 90 mg/g GAE of phenolic content, respectively [46]. Bag et al. reported that the total phenolic contents of *T. chebula*, *T. belerica*, and *E. officinalis* extracted from 70% ethanol were found to be 118.5, 102.6, and 82.7 mg/g GAE [53].

The drug release profile was assessed by determining the total phenolic content in nanotriphala compared to the extract. Polyphenols, including flavonoids, tannins, phenolic acids, and glycosides, are considered the main bioactive compounds in Triphala [54]. Among these, several polyphenols in Triphala have been reported to possess anti-inflammatory activity. As shown in the HPLC chromatogram (Figure S1), fourteen polyphenols were identified, yet several compounds remain unidentified. Therefore, the total phenolic content was subsequently used as a representative measure of the main bioactive compounds in both Triphala and the nanoparticles. The release of total phenolic content from nanotriphala was assessed using a Transwell^®^ chamber. In vitro release data demonstrated that the cumulative amount of phenolic contents released from nanotriphala was 92.79 ± 2.94% within 24 h and completely released within 48 h (101.7 ± 3.3%). The high release rate may be attributed to the high degree of solubility of phenolic content in the nanotriphala matrix. The high extent of phenolic content on the surface of nanoparticles might be due to the formation of a surfactant boundary layer on the nanotriphala surface [36]. The chemical stability of phenolic compounds in nanotriphala stored at 4, 30, and 45 °C gradually decreased over 3 months. The total phenolic content in nanotriphala reduced to 84.3%, 77.8%, and 83.5% when stored at 4, 30, and 45 °C, respectively. These results suggested that phenolic compounds in Triphala in nanotriphala were stable when stored at a temperature up to 45 °C. The rapid degradation of chebulagic acid and chebulinic acid can be attributed to their susceptibility to hydrolyzation and thermal degradation, as noted by Dhingra, et al. [55]. Conversely, the relatively minor degradation of gallic acid is likely due to its inherent heat stability, which makes it less susceptible to temperature-induced breakdown, corroborated by the findings of Pantoja, et al. [56]. This study underscores the importance of evaluating the stability of marker compounds under various conditions, including pH and light exposure. Notably, gallic acid demonstrated the most stability throughout 6-month storage [57]. Martin et al. have shown that gallic acid in the emulsion was stable after storage for 5 months at 38 °C [58]. Galmarini et al. observed that the freeze-dried gallic acid content remained constant when stored in opaque bottles at temperatures of 38 °C for 70 days of storage [59]. Gallic acid, catechin, epicatechin, caffeic acid, and resveratrol remained approximately constant throughout the storage period (38 °C) for 145 days [60]. However, the current study’s scope was limited to room temperature conditions. Future research should extend to a broader range of conditions to provide a more comprehensive understanding of the stability profiles of these bioactive compounds in nanotriphala.

Triphala and its active compound, chebulagic acid and chebulinic acid, have been reported as herbal medicine for controlling COVID-19 disease and have been reported as promising inhibitors of SARS-CoV-2 M^pro^. However, their inhibitory molecular mechanisms remain unexplored [26]. Our study investigated the in vitro inhibitory effects of nanotriphala on CoV2-SP-induced inflammation by determining the inhibition of the final products of the NLRP3 inflammasome pathway at both gene and protein levels on A549 cells. Regarding our in vitro investigation of the CoV2-SP-induced inflammatory mechanism and the therapeutic potential of nanotriphala, gallic acid, chebulagic acid, and chebulinic acid, we selected A549 cells as our cell culture model. This choice is based on previous studies that used ACE2-expressing airway epithelial cells to study SARS-CoV-2 infection and inflammation [8,27]. Specifically, these studies used A549 cells to represent the lower respiratory system or alveolar epithelial cells induced by the CoV2-SP. Therefore, the A549 cells could then activate inflammatory signaling, resulting in the release of pro-inflammatory cytokines. This model has also been used in other research studies as well as in our own previously published studies [35,43,61].

In a previous study, all candidate inflammatory gene expressions (*IL-6*, *IL-1β*, *IL-18*, and *NLRP3*) and cytokine releases (IL-6, IL-1β, and IL-18) in A549 cells were upregulated at 3 h after CoV2-SP induction at a concentration 100 ng/mL [43]. Therefore, pre-treating nanotriphala and its bioactive compounds for at least 3 h in the A549 cell line is necessary to effectively inhibit pro-inflammatory gene expressions in CoV2-SP-induced A549 cells. Moreover, pre-treating cells with nanotriphala for 24 h before induction with CoV2-SP helps the cells reach a steady state. This steady state allows the treatment to exert its effects before experimental induction, ensuring that the conditions are optimal for observing the true effects of the drug. This approach improves the reliability and interpretability of the results [45,62,63].

There is an exaggerated immune response, during SARS-CoV-2 infection, resulting in an uncontrolled release of pro-inflammatory cytokines, a condition known as the “cytokine storm” effect [64]. The NLRP3 inflammasome is a crucial mediator in initiating the cytokine storm associated with COVID-19 [65]. Upon activation by viral components or other danger signals released during infection, when the NLRP3 inflammasome component (comprised of ASC, NLRP3, and Pro-caspase-1) is triggered, the protein complex is assembled, and the inflammasomes cleaved-caspase-1. Subsequently, the NLRP3 inflammasome can lead to the production and release of IL-1β and IL-18, which are potent pro-inflammatory cytokines [66,67]. Our results indicate that nanotriphala exhibited anti-inflammatory properties upon CoV2-SP-induction in A549 lung epithelial cells by inhibiting the NLRP3 inflammasome pathway, leading to suppression at both the gene (*IL-6*, *IL-1β*, *IL-18*, and *NLRP3*) and protein levels (NLRP3, ASC, and Caspase-1) as well as the cleaved-caspase-1 proteins. This led to the suppression of inflammatory cytokines releases such as IL-6, IL-1β, and IL-18. Therefore, it can be inferred that nanotriphala could attenuate the inflammatory responses upon CoV2-SP-induction through the inhibition of the NLRP3 inflammasome pathway in A549 cells. This evidence not only supports the therapeutic potential of nanotriphala but also sheds light on the underlying mechanisms involved in its action, particularly in modulating the NLRP3 inflammasome pathway. In our study, the bioactivity of the Triphala ethanolic extract (unformulated) group was compared with the nanotriphala formulation at the same concentration (300 µg/mL) to evaluate the efficacy of the formulation. The results from cytotoxicity, cytokine secretion, and inflammatory gene expression tests showed no significant differences between the Triphala extract and nanotriphala. Therefore, while the nanotriphala formulation increased the solubility of the Triphala extract, it did not affect its anti-inflammatory property.

This study highlights the anti-inflammatory properties of nanotriphala and its active compounds, chebulagic acid and chebulinic acid, in inhibiting NLRP3-dependent inflammasome activation and cytokine release induced by CoV2-SP in A549 lung cells. Several experiments have reported that the TLR pathway and NLRP3 inflammasome pathway are key targets in the inflammatory response [11,68,69]. Researchers have sought effective compounds that inhibit these pathways to reduce pro-inflammatory cytokines such as IL-6, IL-beta, IL-8, and TNF-alpha, which play important roles in acute respiratory distress syndromes (ARDSs) and are a cause of patient mortality [70]. Nevertheless, further studies on the anti-inflammatory properties of nanotriphala should explore other aspects, such as its effects when induced by the actual virus, its potential for ACE2 inhibition, and its anti-inflammatory effects in animal models. These studies are necessary to fully understand the bioactivity of nanotriphala.

For the further application of nanotriphala in drug delivery to the lungs by inhalation, it must be formulated as an aerosol. Aerosol preparations are stable dispersions or suspensions of solid particles and liquid droplets in a gaseous medium. When administered as an aerosol, the drug is deposited in the airways through gravitational sedimentation, inertial impaction, and diffusion. This method allows nanotriphala to be efficiently and noninvasively delivered to the lung area by inhalation, enabling the active compounds to directly affect lung cells before degradation or metabolism occurs. Pulmonary aerosol can be administered through either nasal or oral inhalation [71]. Overall, the novelty of developing nanoparticles loaded with Triphala extract (nanotriphala) not only enhances the physical and chemical stability of Triphala but also allows for the use of nanotriphala to reduce the effects of the uncontrolled release of pro-inflammatory cytokines, also known as the cytokine storm, caused by SARS-CoV-2 infection. By delivering the active compounds directly to the lungs, nanotriphala can potentially offer a targeted and efficient therapeutic approach with minimized systemic side effects.

## 5. Conclusions

This study demonstrated the development of nanoparticle preparation of Triphala, termed nanotriphala, to enhance its aqueous solubility and to be used for its anti-inflammatory properties against CoV2-SP-induced inflammation in A549 lung cells. Our findings have implications for the management of COVID-19-related inflammation. By demonstrating the efficacy of nanotriphala in attenuating inflammatory responses, our study provides valuable insights into potential therapeutic strategies for mitigating severe COVID-19 symptoms and preventing long-term complications. Further investigations into the anti-inflammatory properties of nanotriphala in normal lung tissues or acute toxicity testing in animal models are warranted, as well as preclinical and clinical studies to evaluate its safety and efficacy in COVID-19 patients. The information obtained from this study could be a valuable source of evidence to support the targeting of the NLRP3 inflammasome pathway in developing preventive strategies for COVID-19-related inflammation.

## Figures and Tables

**Table 1:** The gradient elution of the HPLC system.

Time (min)	1% Acetic Acid in Water pH 2.65	Methanol
0	90	10
10	80	20
15	72	28
20	65	35
25	50	50
26	0	100
30	0	100
32	90	10
40	90	10
50	90	10

**Table 2:** Primer sequences were used in this study for the determination of gene expressions by RT-qPCR analysis [35].

Gene Product	Primer Sequences
*IL-6*	Forward: 5′-ATG AAC TCC TTC ACA AGC-3′ Reverse: 5′-GTT TTC TGC CAG TGC CTC TTT G-3′
*IL-1* *β*	Forward, 5′-TGC TCA AGT GTC TGA AGC AG-3′ Reverse, 5′-TGG TGG TCG GAG ATT CGT AG-3′
*IL-18*	Forward, 5′-TCG GGA AGA GGA AAG GAA CC-3′ Reverse, 5′-TTC TAC TGG TTC AGC AGC CA-3′
*NLRP3*	Forward, 5′-AAC ATG CCC AAG GAG GAA GA-3′ Reverse, 5′-GGC TGT TCA CCA ATC CAT GA-3′
*GAPDH*	Forward, 5′-TCA ACA GCG ACA CCC AC-3′ Reverse, 5′-GGG TCT CTC TCT TCC TCT TGT G-3′

**Table 3:** Identification of phytochemical compounds in nanotriphala by the HPLC technique.

Standard Compounds	Conc. (mg/100 mg)
Gallic acid	1.19
Chebulinic acid	0.98
Chebulagic acid	0.42
Ascorbic acid	0.27
Maleic acid	0.03
Ellagic acid	<0.0001
Rutin	<0.0001
Resveratrol	<0.0001
Quercetin	<0.0001
Kaempferol	<0.0001
2,4-Dihydroxy benzoic acid	<0.0001
Catechin	<0.0001
Epicatechin	<0.0001
Quercitrin	<0.0001

## Data Availability

Data are contained within the article and Supplementary Materials.

## Supplementary Materials

The following supporting information can be downloaded at: https://www.mdpi.com/article/10.3390/pharmaceutics16060751/s1, Table S1: Solubility test results of Triphala ethanolic extract (10 and 20 mg/mL) in various solvents; Table S2: Water solubility testing of Triphala extract, nanotriphala, and blank control (1, 5, 10 and 20 mg/mL); Figure S1: The HPLC Profile of reference standards and nanotriphala. HPLC chromatogram of reference standards, 1 = Ascorbic acid, 2 = Maleic acid, 3 = Gallic acid, 4 = 2,4-Dihydroxybenzoic acid, 5 = Catechin, 6 = Epicatechin, 7 = Chebulagic caid, 8 = Rutin, 9 = Ellagic acid, 10 = Chebulinic acid, 11 = Quercitrin, 12 = Resveratrol, 13 = Quercetin, and 14 = Kaempferol at a concentration of 0.02 mg/mL for each standard (A). HPLC chromatogram of nanotriphala at concentration 10 mg/mL (B). HPLC chromatogram of nanotriphala at concentration 20 mg/mL spike with reference standards at concentration 0.02 mg/mL (C). The HPLC chromatogram were evaluated using reversed-phase C18 column. The detection wavelength was 295 nm. The flow rate was set to 1.0 mL/min; Figure S2: Concentration of gallic acid, chebulagic acid, chebulinic acid in nanotriphala over 6-month period: ** *p* < 0.05; Figure S3: Cell viability of nanotriphala and blank control (A), Triphala extract (B), gallic acid (C), chebulagic acid (D), and chebulinic acid (E) on A549 cells. Cells were treated with Triphala extract, nanotriphala, and blank control (0–300 μg/mL), and active compounds (gallic acid, chebulagic acid, and chebulinic acid) (0–50 μg/mL) for 24 and 48 h. Cell survival was determined using an SRB assay. Data are presented as mean ± S.D. values of three independent experiments, *** *p* < 0.001 compared with the control group; Figure S4: Inhibitory effects of Triphala extract, nanotriphala, and blank control on the pro-inflammatory cytokine secretion in CoV2-SP-induced A549 cells. A549 cells were pre-treated with Triphala extract, nanotriphala, and blank control (300 µg/mL) for 24 h. Then, the cells were exposed to CoV2-SP (100 ng/mL) for 3 h. The IL-6 (A), IL-1β (B), and IL-18 secretions (C) in the culture supernatant were examined by ELISA. The CoV2-SP-induced A549 cells are presented as 100%. Data are presented as mean ± S.D. values of three independent experiments, ^ns^ non-significant (*p* > 0.05) compared nanotriphala with Triphala extract at the same concentration; Figure S5: Inhibitory effects of Triphala extract, nanotriphala, and blank control on the *IL-6* (A), *IL-1β* (B), *IL-18* (C), and *NLRP3* gene expressions (D) in CoV2-SP-induced A549 cells. A549 cells were pre-treated with Triphala extract, nanotriphala, and blank control (300 µg/mL) for 24 h. Then, the cells were exposed to CoV2-SP (100 ng/mL) for 3 h. The mRNA expressions were determined using RT-qPCR. Data are presented as mean ± S.D. values of three independent experiments, ^ns^ non-significant (*p* > 0.05) compared nanotriphala with Triphala extract at the same concentration.
