# Supplementary material for: Inhibition of SARS-CoV-2-Induced NLRP3 Inflammasome-Mediated Lung Cell Inflammation by Triphala-Loaded Nanoparticle Targeting Spike Glycoprotein S1
Source: Pharmaceutics. 2024 Jun 2;16(6):751. doi: 10.3390/pharmaceutics16060751 (PMC11206841; doi:10.3390/pharmaceutics16060751)
Supplement: Supplementary file 1 [file pharmaceutics-16-00751-s001.zip › pharmaceutics-2994880-supplementary.pdf]

Supplementary information

Table S1. Solubility test results of Triphala ethanolic extract (10 and 20 mg/mL) in various solvents.

	Conc. (mg/mL)	Hexane	Dichloro-methane	Ethyl acetate	Ethanol	Water	DMSO
Triphala extract	10	0 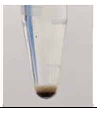	0 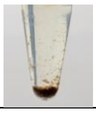	0 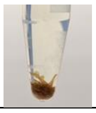	+1 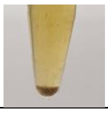	+1 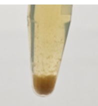	+3 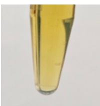
	20	0 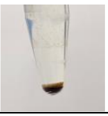	0 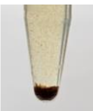	0 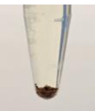	+1 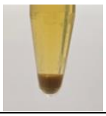	+1 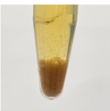	+3 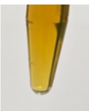

+3 = Fully soluble, +2 = Partially soluble, +1 = Slightly soluble, and 0 = Insoluble, at room temperature.
Abbreviation: DMSO: dimethyl sulphoxide

Table S2. Water solubility testing of Triphala extract, nanotriphala, and blank control (1, 5, 10 and 20 mg/mL).

Conc. (mg/mL)	Triphala extract	Nanotriphala	Blank control
1	+3 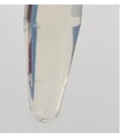	+3 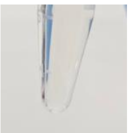	+3 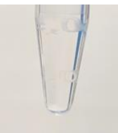
5	+2 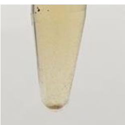	+3 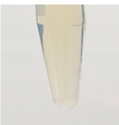	+3 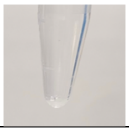
10	+1 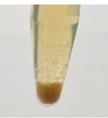	+3 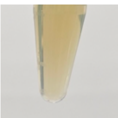	+3 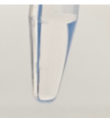
20	+1 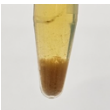	+3 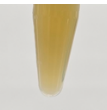	+3 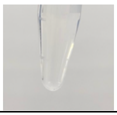

+3 = Fully soluble, +2 = Partially soluble, +1 = Slightly soluble, and 0 = Insoluble, at room temperature.

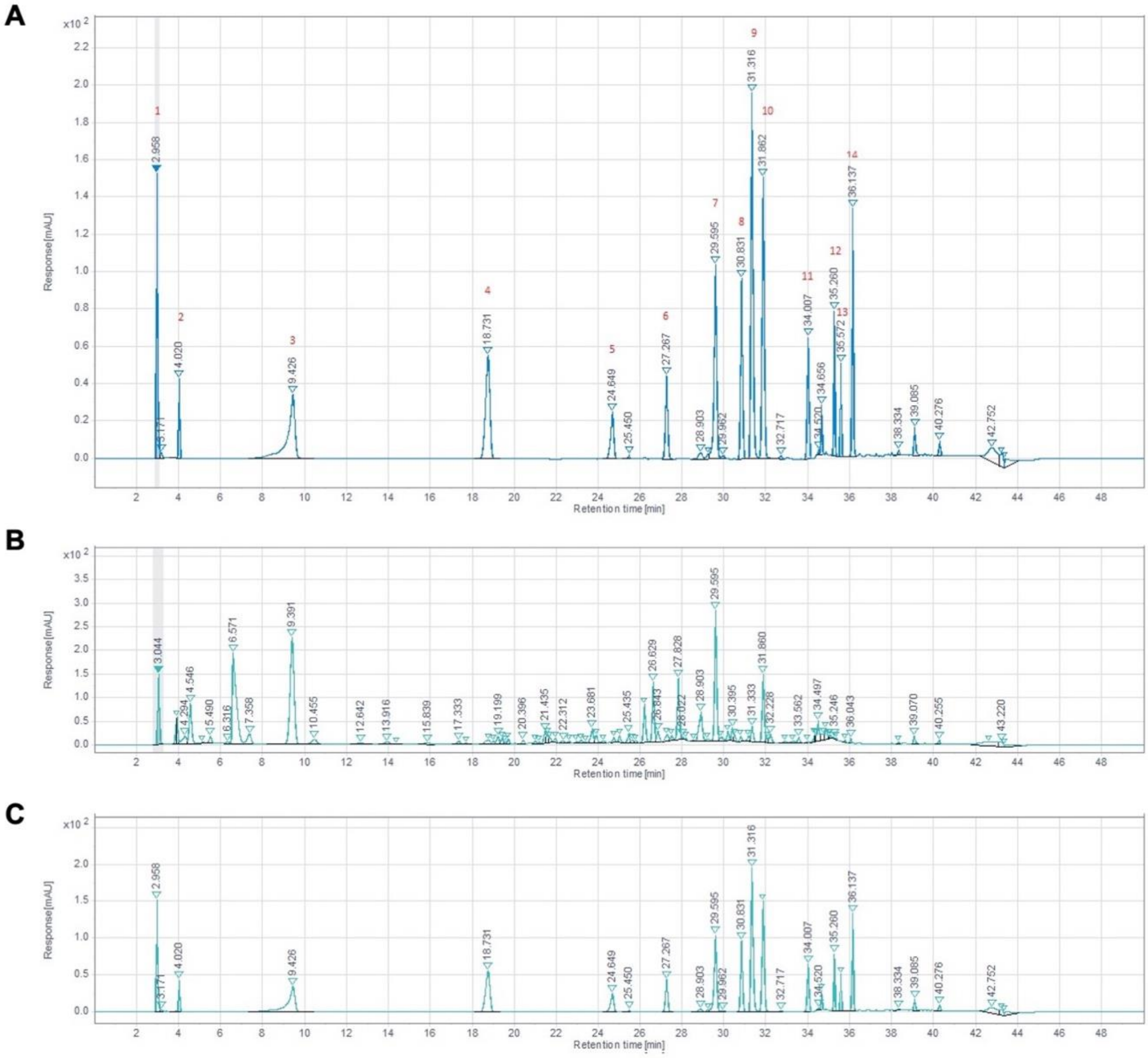

Figure S1. The HPLC Profile of reference standards and nanotriphala. HPLC chromatogram of reference standards, 1 = Ascorbic acid, 2 = Maleic acid, 3 = Gallic acid, 4 = 2,4-Dihydroxybenzoic acid, 5 = Catechin, 6 = Epicatechin, 7 = Chebulagic caid, 8 = Rutin, 9 = Ellagic acid, 10 = Chebulinic acid, 11 = Quercitrin, 12 = Resveratrol, 13 = Quercetin, and 14 = Kaempferol at a concentration of 0.02 mg/mL for each standard (A). HPLC chromatogram of nanotriphala at concentration 10 mg/mL (B). HPLC chromatogram of nanotriphala at concentration 20 mg/mL spike with reference standards at concentration 0.02 mg/mL (C). The HPLC chromatogram were evaluated using reversed-phase C18 column. The detection wavelength was 295 nm. The flow rate was set to 1.0 mL/min.

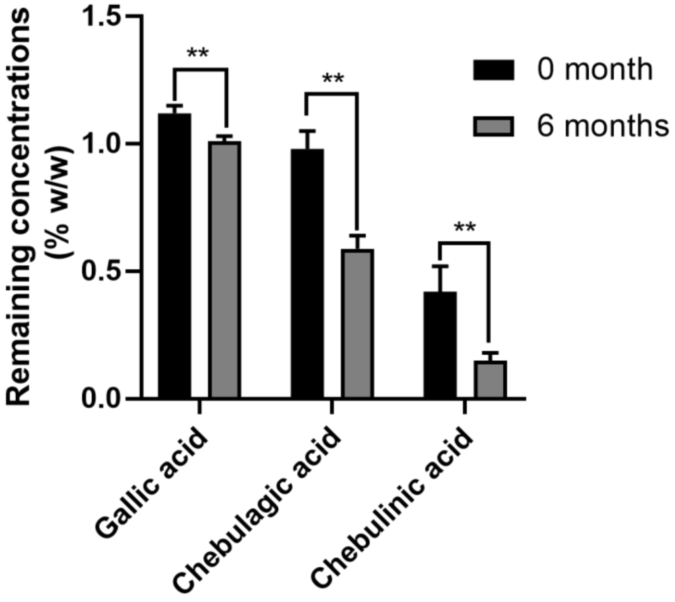

Figure S2. Concentration of gallic acid, chebulagic acid, chebulinic acid in nanotriphala over 6-month period: ** $p < 0.05$.

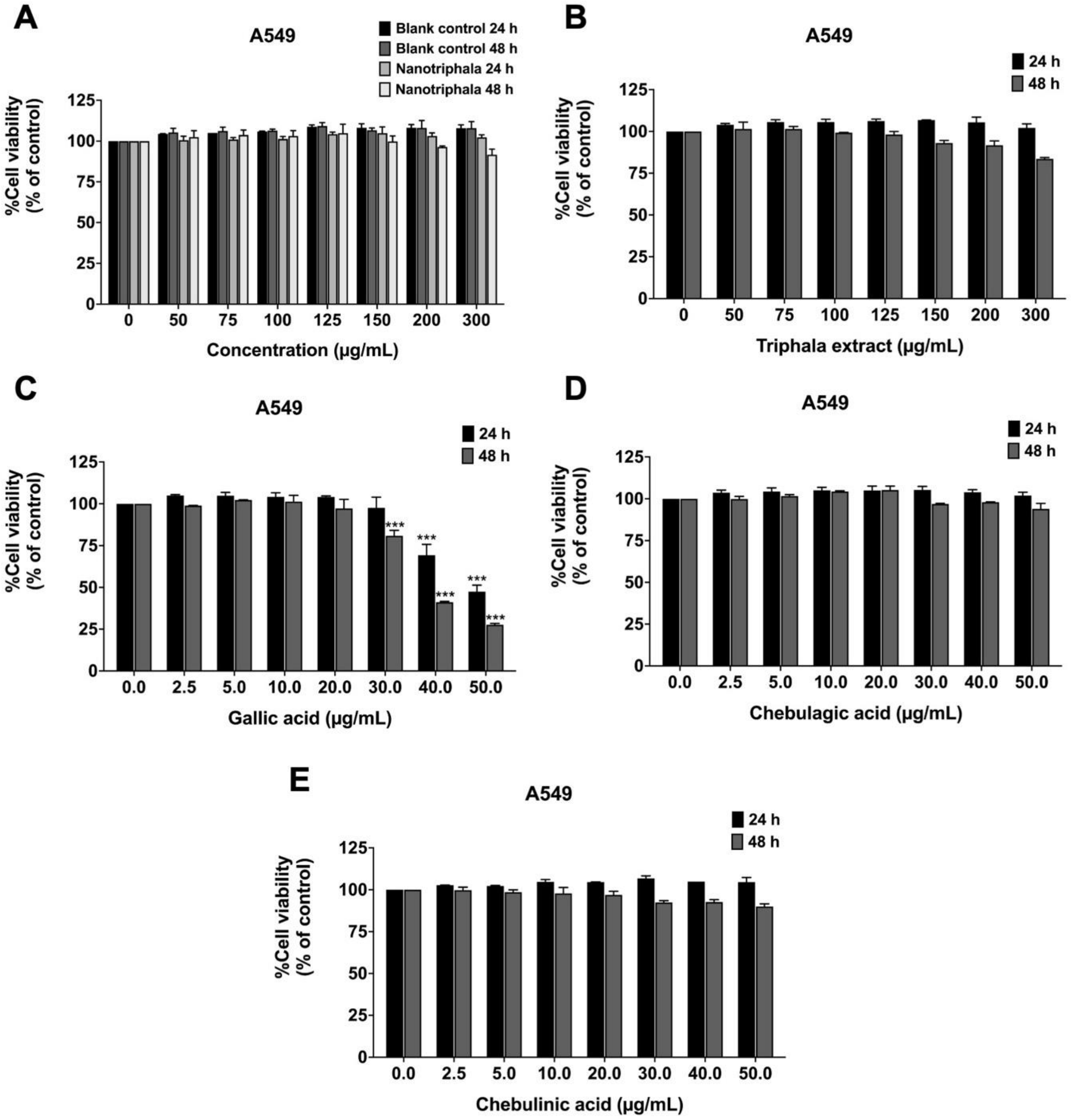

Figure S3. Cell viability of nanotriphala and blank control (A), triphala extract (B), gallic acid (C), chebulagic acid (D), and chebulinic acid (E) on A549 cells. Cells were treated with triphala extract, nanotriphala, and blank control (0–300 μg/mL), and active compounds (gallic acid, chebulagic acid, and chebulinic acid) (0-50 μg/mL) for 24 and 48 h. Cell survival was determined using an SRB assay. Data are presented as mean ± S.D. values of three independent experiments, *** $p < 0.001$ compared with the control group.

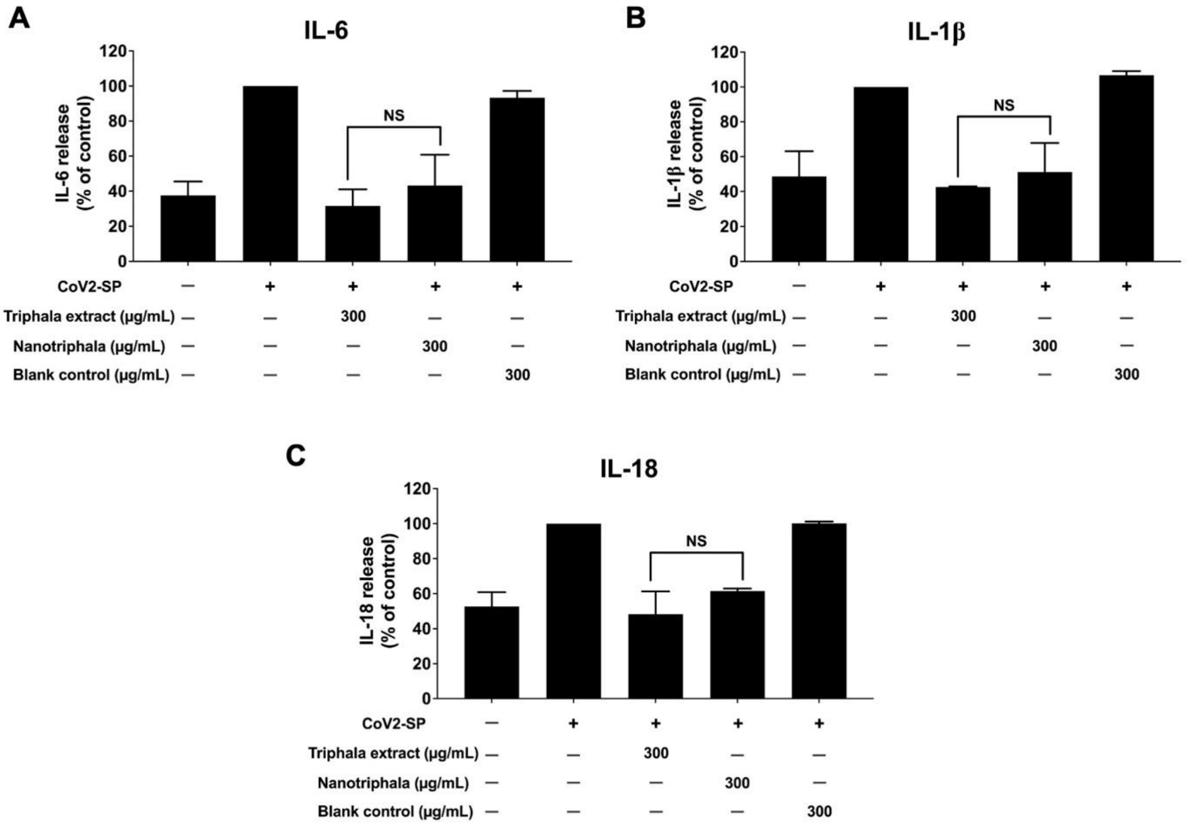

Figure S4. Inhibitory effects of triphala extract, nanotriphala, and blank control on the pro-inflammatory cytokine secretion in CoV2-SP-induced A549 cells. A549 cells were pre-treated with triphala extract, nanotriphala, and blank control (300 µg/mL) for 24 h. Then, the cells were exposed to CoV2-SP (100 ng/mL) for 3 hours. The IL-6 (A), IL-1β (B), and IL-18 secretions (C) in the culture supernatant were examined by ELISA. The CoV2-SP-induced A549 cells are presented as 100%. Data are presented as mean ± S.D. values of three independent experiments, ^{ns} non-significant ($p > 0.05$) compared nanotriphala with triphala extract at the same concentration.

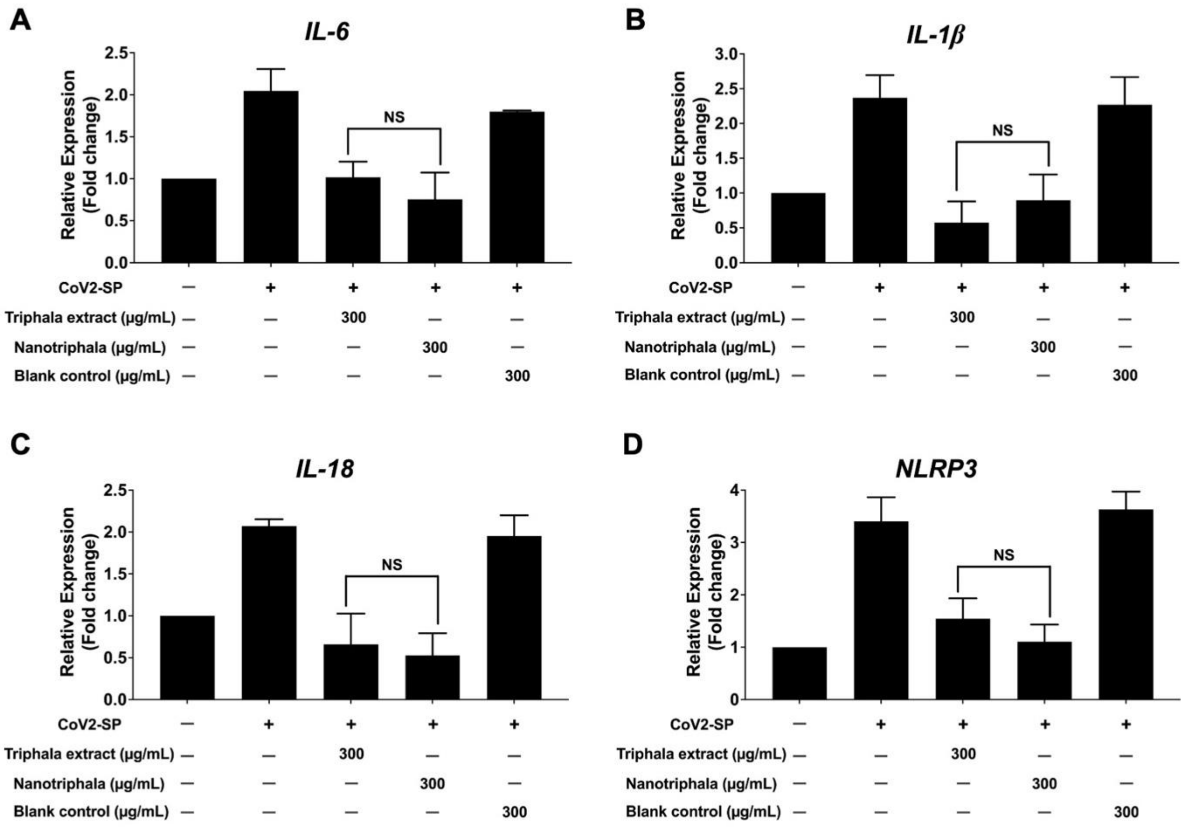

Figure S5. Inhibitory effects of triphala extract, nanotriphala, and blank control on the *IL-6* (A), *IL-1β* (B), *IL-18* (C), and *NLRP3* gene expressions (D) in CoV2-SP-induced A549 cells. A549 cells were pre-treated with triphala extract, nanotriphala, and blank control (300 μg/mL) for 24 h. Then, the cells were exposed to CoV2-SP (100 ng/mL) for 3 hours. The mRNA expressions were determined using RT-qPCR. Data are presented as mean ± S.D. values of three independent experiments, ^{ns} non-significant ($p > 0.05$) compared nanotriphala with triphala extract at the same concentration.
